# Phytochemical analysis of Vietnamese propolis produced by the stingless bee *Lisotrigona cacciae*

**DOI:** 10.1371/journal.pone.0216074

**Published:** 2019-04-24

**Authors:** Kristina Georgieva, Milena Popova, Lyudmila Dimitrova, Boryana Trusheva, Le Nguyen Thanh, Diep Thi Lan Phuong, Nguyen Thi Phuong Lien, Hristo Najdenski, Vassya Bankova

**Affiliations:** 1 Institute of Organic Chemistry with Centre of Phytochemistry, Bulgarian Academy of Sciences, Sofia, Bulgaria; 2 Department of Infectious Microbiology, The Stephan Angeloff Institute of Microbiology, Bulgarian Academy of Sciences, Sofia, Bulgaria; 3 Institute of Marine Biochemistry and Graduate University of Science and Technology, Vietnam Academy of Science and Technology, Hanoi, Vietnam; 4 Department of Chemistry, Quy Nhon University, Binh Dinh, Vietnam; 5 Institute of Ecology and Biological Resources, Vietnam Academy of Science and Technology, Hanoi, Vietnam; Universitat Leipzig, GERMANY

## Abstract

Propolis produced by the stingless bee *Lisotrigona cacciae* was studied for the first time. Using different chromatographic procedures, a total of eighteen constituents (phenols and triterpenes) were isolated, among which flavane **1**, homoisoflavanes **2**–**4**, and xanthones **5** and **6** were new for propolis. Propolis extract was also characterized by gas chromatography/mass spectrometry and other fifteen constituents were identified. The xanthone *α*-mangostin (**8**) demonstrated significant activity against *Staphylococcus aureus* with MIC and MBC 0.31 μg/ml, followed by 7,4'-dihydroxy-5-methoxy-8-methylflavane (**1**) with MIC 78 μg/ml and MBC 156 μg/ml. 10,11- Dihydroxydracaenone C (**4**), a component bearing *ortho*-hydroxyl groups, was the only compound displaying radical scavenging ability. Triple botanical origin of the sample was defined, consisting of *Dracaena cochinchinensis*, *Cratoxylum cochinchinense* and *Mangifera indica*. *D*. *cochinchinensis* is a new resin source of propolis.

## Introduction

Propolis is a valuable beehive product containing plant secretions and beeswax. It is well known as a remedy with a wide range of biological and pharmacological properties, such as antibacterial, antioxidant, immunostimulating, antiviral, etc. [1,2]. Propolis chemistry depends on the geographical origin, plant species, and bee species, and thus various constituents contribute to its bioactivity [3]. Because of its chemical diversity, propolis has been classified into types based on the plants that bees have chosen as resin sources. At present, the majority of scientific information concerns propolis produced by the honey bee *Apis mellifera* (tribe Apini), which inhabit almost all ecosystems of the world, and over twenty propolis types have been formulated [3,4]. In tropical and southern subtropical regions, however, the native bee species are stingless bees (tribe Meliponini), which are also key pollinators and producers of beneficial honey, wax and propolis [5,6].

Unlike honey bees, stingless bees are a diverse group, and more than 500 species have been described [7]. Despite the traditional use of stingless bee products, a few studies concerning propolis have been published, particularly for propolis originating from Mainland Southeast Asia. Moreover, in this region bee species with unique behavior have been recorded such as the minute lachryphagous species of a rare genus *Lisotrigona* [8–11]. These bees have been investigated from biological and ecological point of view [11–14], while the products they manufacture are only scarcely analysed [15]. The first data on *Lisotrigona* spp. propolis appeared in 2018 [16–18]. Xanthones and triterpenes, new propolis constituents, were found in a sample collected by *Lisotrigona furva* in Vietnam.

In the present article, we report results of the phytochemical analysis of Vietnamese propolis produced by *Lisotrigona cacciae*, its antimicrobial and antioxidant activity. Using different chromatographic procedures, a total of 18 constituents were isolated, among which flavane **1**, homoisoflavanes **2**–**4**, and xanthones **5** and **6** were found in propolis for the first time. Crude propolis extract was also characterized by GC/MS after silylation, and other 15 constituents were identified. The botanical origin of the sample was defined, and a new plant source *Dracaena cochinchinensis* was suggested.

## Materials and methods

### Ethics statement

No specific permits were required for the described field studies. The field studies did not involve endangered or protected species, and were conducted on private land with owner permission.

### General data

NMR spectra were recorded on a Bruker AVANCE II+ 600 NMR spectrometer operating at 600 MHz (150 MHz for ^13^C). Optical rotation was measured on a Jasco P-2000 polarimeter. Vacuum liquid chromatography (VLC) was performed on Silica gel 60H (Merck, 15 μm). Column chromatography (CC) was performed on Silica gel 60 (Merck, 63–200 μm) normal phase, and Sephadex LH-20 (Pharmacia Fine Chemicals, 25–100 μm). Low pressure liquid chromatography (LPLC) was carried out with LiChroprep Si 60 Merck column (40–63 μm). Preparative thin-layer chromatography (prep. TLC) was performed on silica gel 60 F_254_ glass plates (Merck, 20 x 20 cm; 0.25 mm). Detection of the spots was achieved under UV light at 254 and 366 nm, and by spraying with vanillin in sulfuric acid, followed by heating at 100°C. All solvents used were of analytical grade.

### Propolis sample

The propolis sample was collected by scraping from stingless bees’ hives of *L*. *cacciae* from Binhdinh province in Vietnam’s South Central Coast region in July, 2017. The stingless bee species was identified by Dr. Nguyen Thi Phuong Lien, Department of Insect Ecology, Institute of Ecology and Biological Resources, Vietnam Academy of Science and Technology. The sample was characterized by a deep red color. The flow chart of the sample analysis is shown on S1 Fig.

### GC/MS analysis

The procedure of the GC/MS analysis was similar to the one described previously [19]. The propolis sample was grated after cooling, and extracted with 70% ethanol (1:10, w/v) at room temperature (2 x 24 h). A part (20 ml) of the crude extract was evaporated to dryness *in vacuo*. About 5 mg of the extract were mixed with 50 μl of dry pyridine and 75 μl of N,O-bis(trimethylsilyl)trifluoroacetamide (BSTFA) and heated at 80°C for 20 min. The reference compounds, isolated from the sample, were subjected to the same silylation procedure as about 1 mg of the compound was mixed with 10 μl of dry pyridine and 15 μl of BSTFA. The GC/MS analysis of the silylated samples (TMS derivatives) was performed on Agilent 7820 GC System/5977B MSD instrument equipped with a 60 m long, 0.25 mm i.d., and 0.25 μm film thickness DB-5MS UI capillary column. The temperature was programmed from 100 to 325°C at a rate of 5°C/min, and a 30 min hold at 325°C. Helium was used as a carrier gas at a flow rate of 0.8 ml/min. The split ratio was 1:50, the injector temperature 300°C, the interface temperature 300°C, and the ionization voltage 70 eV. The identification of the compounds was performed using commercial libraries, literature data and/or comparison with mass spectra of reference compounds.

### Extraction and isolation

Raw propolis sample (160 g) was grated after cooling and extracted with 70% ethanol (1:10, w/v) at room temperature (2 x 24 h). The combined ethanol extracts were concentrated and subjected to liquid-liquid extraction successively with petroleum ether (PE, 3 times) and diethyl ether (DEE, 3 times) to give 3 g PE and 2.5 g DEE dry residue. PE extract (2.5 g) was subjected to CC on Sephadex LH-20, eluted with CH_3_OH, and 10 fractions were obtained (A-J). Fraction A (1.3 g) was subjected to VLC on silica gel, eluted with PE–EtOAc (1:0 to 0:1), and 11 subfractions were obtained (A1-A11). Subfraction A1 (58 mg) was subjected to LPLC eluted with PE:Acetone to give cycloartenone **10** (17.7 mg) [20]. Fraction C (40 mg) was purified by prep. TLC with CH_2_Cl_2_:EtOAc (10:1) to yield lupeol **11** (4.3 mg) [21] and an inseparable mixture of resorcinols **12a-g** (6.1 mg) [22]. Fraction F yielded cochinchinone A **7** (36.8 mg) [23]. From fraction H (52 mg), a mixture of 3-geranyloxy-1,7-dihydroxyxanthone (cochinchinone G) **5** and 7-geranyloxy-1,3-dihydroxyxanthone **6** (1.2 mg) [23] were obtained by prep. TLC with PE:EtOAc (3:2). Fraction J (31.1 mg) was purified by prep. TLC with CH_2_Cl_2_:EtOAc as a mobile phase (20:1) to yield *α*-mangostin **8** (15.2 mg) [24].

DEE extract (2 g) was subjected to VLC on silica gel using PE:EtOAc (1:0 to 0:1) as a mobile phase. Twenty two fractions were obtained (A-V). Fractions D and E were combined (242 mg) and subjected to CC on Sephadex LH-20, eluted with CH_3_OH to give 7 subfractions D1– D8. From subfractions D5 (80 mg), after LPLC eluted with CHCl_3_:CH_3_OH (1:0 to 9:1), seven subfractions (D5-1 - D5-7) were obtained. Subfraction D5-2 (39,2 mg) and D5-4 (19,2 mg) were purified by prep. TLC with CHCl_3_-EtOAc (9:1) to yield again compound **8** (6.0 mg), and (2*R*)-7,4'-dihydroxy-5-methoxy-8-methylflavane **1** (8.9 mg) ${\left[\alpha \right]}_{D}^{20}$ +130.5° (*c* 0.26, CH_3_OH) [25], respectively. From fraction F (180 mg) after subsequent usage of silica gel CC, eluted with CHCl_3_:CH_3_OH (1:0 to 9:1) and prep. TLC with PE:EtOAc (7:3), a (3*R*)-7,4'-dihydroxyhomoisoflavane **2** (18 mg) ${\left[\alpha \right]}_{D}^{20}$ +79.2° (*c* 0.26, CH_3_OH) [26,27] was isolated. Fraction G (240 mg) was subjected to CC on Sephadex LH-20, eluted with CH_3_OH to give 9 subfractions (G1-G7). Subfraction G5 (31 mg) was separated by prep. TLC with CHCl_3_:EtOAc (8:2) to yield garcinone B **9** (1.5 mg) [28], again compounds **1** (1.8 mg) and **2** (2 mg), and (3*S*)-7,4'-dihydroxy-5-methoxyhomoisoflavane **3** (9.3 mg) ${\left[\alpha \right]}_{D}^{21}$ -44° (*c* 0.35, CH_3_OH) [27,29]. Fractions O-R (151 mg) were washed with CHCl_3_, and white crystals of a 10,11-dihydroxydracaenone C **4** (14.2 mg) ${\left[\alpha \right]}_{D}^{21}$ -457° (*c* 0.12, CH_3_OH) [30] were obtained.

The compounds structures were elucidated using 1D and 2D NMR experiments, optical rotation data, and literature data comparison. The spectra and solvents used are indicated in the Supporting information (S2–S12 Figs).

### Antimicrobial activity

Antimicrobial activity against *Staphylococcus aureus* SAIM 209 (collection of the Stephan Angeloff Institute of Microbiology, Bulgaria), *Escherichia coli* SAIM WF+ and *Candida albicans* SAIM 562 was evaluated in triplicate by the broth microdilution method according to Clinical Laboratory Standard Institute (CLSI) procedures [31] and as published before [32]. The bacterial inoculums with concentration 10^5^ CFU/ml were added to microtitre trays containing Muller Hinton broth (MHB) loaded with extract or isolated compounds with concentrations in the range of 0.039 to 2.5 mg/ml. Plates were incubated at 37°C for 18 h. The negative control was prepared by spreading 10 μl of the inoculation-suspension on a nutrient agar plate and incubated at 37°C overnight. Minimal inhibitory concentration (MIC) was determined visually as the lowest concentration without visible growth. Minimal bactericidal concentration (MBC) was determined by overnight incubation on Muller Hinton agar (MHA) of 100 μl from the untreated control and samples treated with ½ x MIC, MIC and 2 x MIC for further 18 h at 37°C. MBC was read as concentrations where no bacterial growth occurred on the agar plates. The antibiotics Gentamicin and Amphotericin B were used as positive controls against bacteria and fungi, respectively.

### DPPH radical scavenging activity

2,2-Diphenyl-1-picrylhydrazyl (DPPH) radical scavenging activity was evaluated by colorimetric procedure [33]. In brief, 100 μl of the 70% EtOH extract and individual compounds at five concentrations (62.5; 125; 250; 500; 1000 μg/ml) were added to 2 ml of 100 μM DPPH ethanol solution. After 30 min, the absorbance at 517 nm was measured. The free radical scavenging activity was determined by comparison with the absorbance of blank (100%), containing only DPPH in ethanol. A graph plot percentage inhibition against concentration was used to calculate the concentration of the tested samples providing 50% inhibition (IC_50_). Caffeic acid was used as a positive control (6.25, 12.5; 25; 50; 100 μg/ml).

## Results and discussion

### Phytochemical analysis

**Fig 1:**
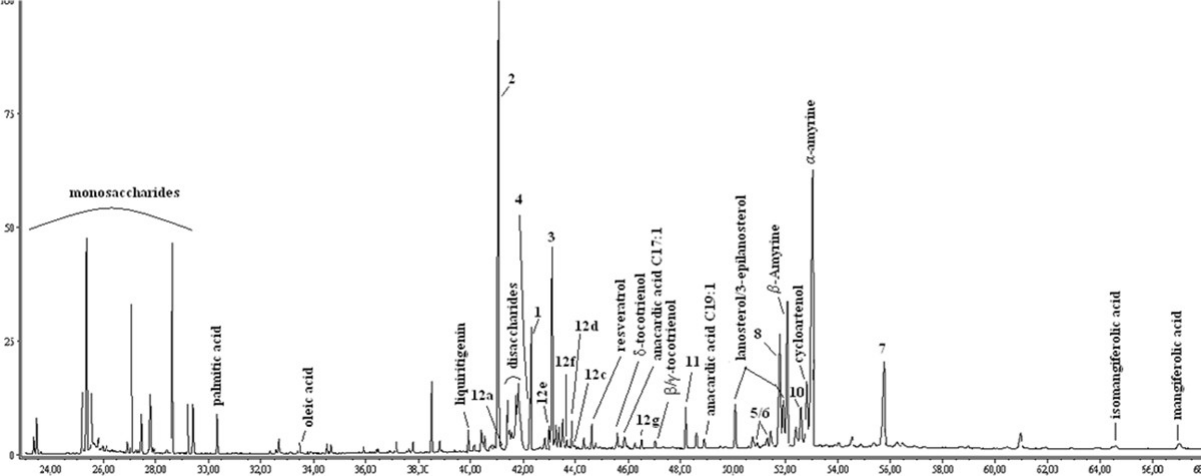
GC chromatogram of 70% ethanol propolis extract. The numbers of compounds identified correspond to those of isolated compounds.

Propolis is a resinous material with chemical diversity, which leads to formulation of propolis types. Most of the types formulated are well characterized by various analytical techniques, including GC/MS after silylation. The trends in propolis research using hyphenated techniques provide valuable information in respect to rapid identification of already known propolis constituents, and thus of propolis type dereplication [4,34]. That is why, the crude extract (70% ethanol) of *L*. *cacciae* propolis was firstly subjected to GC/MS analysis. Besides sugars and fatty acids, common propolis constituents, presence of alk(en)yl resorcinols, anacardic acids and triterpenes (Fig 1; Table 1) typical for propolis originating from *Mangifera indica* (Anacardiaceae) plants [35–38] was revealed. However, compounds corresponding to some of the most prominent peaks in the total ion current (TIC) chromatogram remained unidentified, which provoked us to proceed with isolation and identification of individual constituents.

**Table 1:** Constituents identified in 70% ethanol propolis extract by GC/MS (TMS derivatives).

Component	TIC, %	Component	TIC, %
**Alk(en)ylresorcinols**^a^	**1.4**	**Flavonoids**	**18.7**
Alkylresorcinol C_15_H_31_ (**12a**)	Tr.	7,4'-Dihydroxyflavanone (liquiritigenin)	0,5
Alkylresorcinol C_15_H_29_ (**12b**)	Tr.	(3*R*)-7,4'-Dihydroxyhomoisoflavane (**2**)^b^	10,7
Alkylresorcinol C_17_H_35_ (**12c**)	Tr.	(2*R*)-7,4'-Dihydroxy-5-methoxy-8-methylflavane (**1**)^b^	3,4
Alkylresorcinol C_17_H_33_ (**12d**)	0.6	10,11-Dihydroxydracaenone C (**4**)^b^	
Alkylresorcinol C_17_H_31_ (**12e**)	0.6	(3*S*)-7,4'-Dihydroxy-5-methoxyhomoisoflavane (**3**)^b^	4,1
Alkylresorcinol C_17_H_29_ (**12f**)	Tr.	**Xanthones**	**8.1**
Alkylresorcinol C_19_H_37_ (**12g**)	0.2	3-Geranyloxy-1,7-dihydroxyxanthone (**5**)	4.7
**Anacardic acids**	**0.5**	7-Geranyloxy-1,3-dihydroxyxanthone (**6**)	3.4
Anacardic acid C_17_H_33_	0.3	*α*-Mangostin (**8**)^b^	Tr.
Anacardic acid C_19_H_37_	0.2	Cochinchinone A (**7**)^b^	Tr.
**Triterpenes**	**26.6**	**Other phenols**	**1.0**
*α*-Amyrine	13.4	Resveratrol	0.5
*β*-Amyrine	4.8	*δ*-Tocotrienol	0.3
Cycloartenol	3.0	*β/γ*-Tocotrienol	0.2
Cycloartenone (**10**)	1.5	**Fatty acids and esters**	**0.9**
Lupeol (**11**)	1.0	Palmitic acid	0.7
Mangiferolic acid	Tr.	Oleic acid	0.2
Isomangiferolic acid	Tr.	**Sugars**	**24.2**
Lanosterol	1,3	Monosaccharides	17.3
Lanosterol (3-epi)	1,6	Disaccharides	6.9

Tr., traces (<0.1%TIC).

^a^Identified by comparison with literature data and authentic sample (after isolation).

^b^Identified by comparison with authentic samples (after isolation).

**Fig 2:**
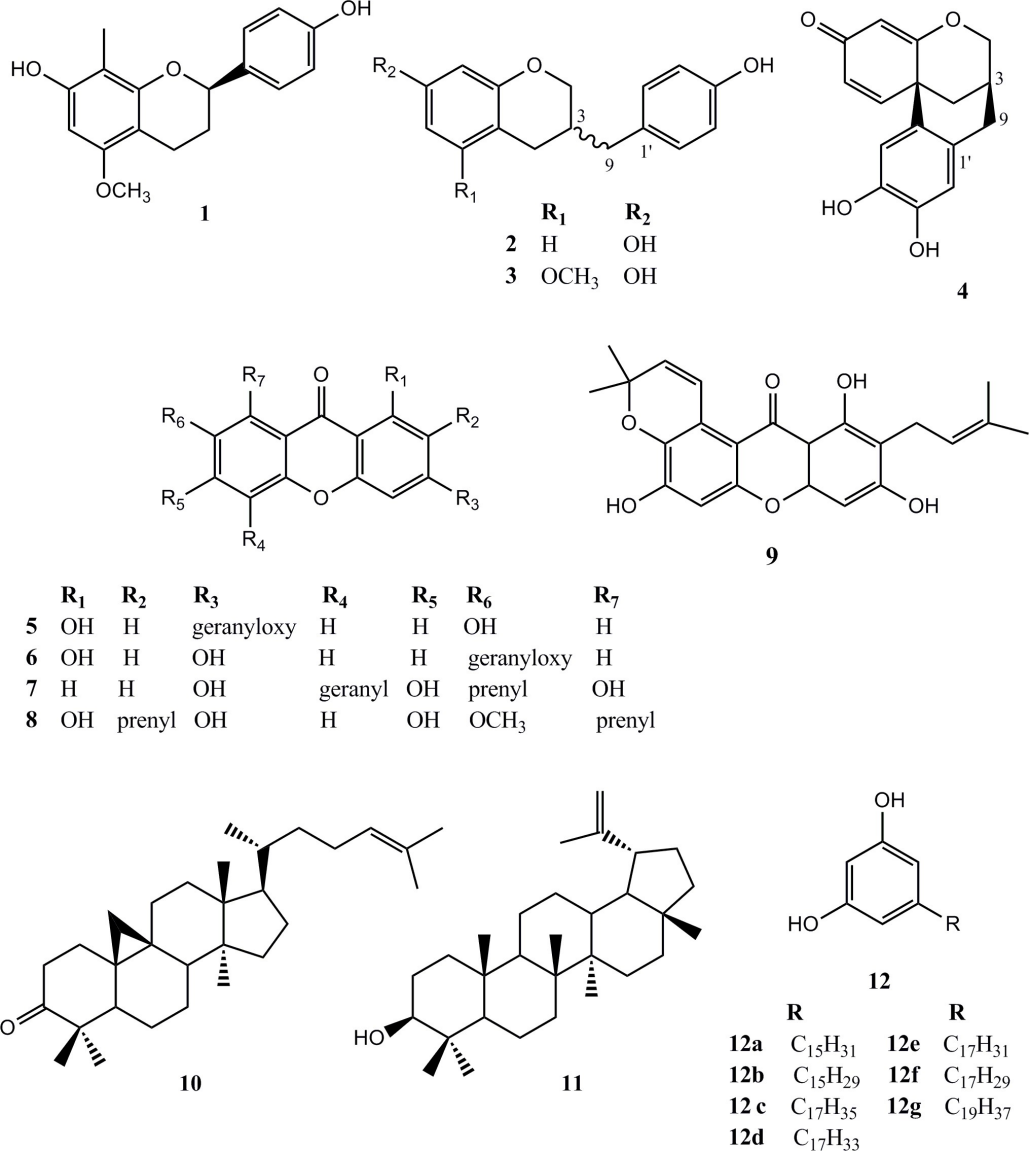
Chemical structures of isolated compounds.

The crude propolis extract was extracted successively with PE and DEE. After repeated CC on silica gel and Sephadex, and prep. TLC flavanes, xanthones, triterpenes and a mixture of alk(en)yl recorcinols were isolated. The structures (Fig 2) were elucidated as: (2*R*)-7,4'-dihydroxy-5-methoxy-8-methylflavane **1**, (3*R*)-7,4'-dihydroxyhomoisoflavane **2**, (3*S*)-7,4'-dihydroxy-5-methoxyhomoisoflavane **3**, 10,11-dihydroxydracaenone C **4**, a mixture of 3-geranyloxy-1,7-dihydroxyxanthone (cochinchinone G) **5** and 7-geranyloxy-1,3-dihydroxyxanthone **6**, 2,6,8-trihydroxy-5-geranyl-7-prenylxanthone (cochinchinone A) **7**, *α*-mangostin **8,** garcinone B **9**, cycloartenone **10**, lupeol **11**, and a mixture of alk(en)yl resorcinols **12** by means of NMR (1D and 2D) experiments, optical rotation data, and literature data comparison. Mixture **12** was additionally characterized and confirmed by GC/MS as consisting of resorcinols **12a-g**. Among the isolated compounds, six (**1**–**6**) were found in propolis for the first time.

Further, selected isolated compounds were used as standards and subjected to GC/MS analysis in order to obtain information for their mass spectral characteristics as TMS derivatives, and then to recognize them in the TIC chromatogram. The data obtained (Table 2) revealed that the fragmentation pathways of the flavonoids **1**–**4** are in accordance with those proposed by Su et al. [39] and Chen et al. [40], who studied Chinese dragon's blood and its constituents by means of IT-TOF HRMS^n^ and UHPLC-QTOF-MS/MS, respectively. In the mass spectra of the homoisoflavans **2** and **3**, the molecular ion was the most prominent peak (base peak), followed by B-ring and AC-rings fragments due to C_3_–C_9_ and C_9_–C_1’_ bond cleavages, which have been regarded as diagnostic for this type of flavonoids [40,41]. Compounds **4** and **1** gave fragmentation patterns identical to those of the homoisoflavanes and flavan-3-ols [42], respectively. The MS spectra of prenyl **8** and geranyl **7** xanthones showed a low intensity molecular ion peak and a base peak [M-15]^+^. Fragment ion [M-43]^+^ due to the subsequent loss of carbonyl group (M-CH_3_-CO) was also detected in both molecules. All these results could be useful in respect to further dereplication purposes, having in mind that the GC/MS (after silylation) is a rapid and very often used analytical technique for metabolite profiling of propolis and plant resins.

**Table 2:** Mass spectral data of isolated compounds (GC/MS, TMS derivatives).

Compound	[M]^+^, *m/z* (%)	Fragments, *m/z* (%)
(2*R*)-7,4'-Dihydroxy-5-methoxy-8-methylflavane (**1)**	430 (100)	415 (29), 399 (12), 357 (10), 251 (8), 238 (95), 223(31), 207 (58), 192 (20), 165 (5), 73 (98)
(3*R*)-7,4'-Dihydroxyhomoisoflavane (**2**)	400 (100)	385 (12), 233 (18), 220 (31), 195 (8), 179 (69), 165 (10), 73 (60)
(3*S*)-7,4’-Dihydroxy-5-methoxyhomoisoflavane (**3**)	430 (100)	415 (16), 264 (15), 250 (50), 225 (9), 179 (45), 165 (7), 73 (74)
10,11-Dihydroxydracaenone C (**4**)	414 (100)	399 (23), 357 (15) 341 (2), 267 (4), 253 (4), 179 (5), 147 (2), 73(44)
Cochinchinone A (**7**)	664 (4)	649 (100), 621 (6), 579 (32), 565 (24), 539 (9), 511 (18), 491 (16), 73 (26)
*α*-Mangostin (**8**)	626 (15)	611 (100), 595 (4), 583 (41), 553 (13), 73 (53)

After the compounds were characterised as TMS derivatives, we succeeded in their reliable identification in TIC chromatogram. All six compounds corresponded to prominent peaks with relative abundance of 3–11% of TIC (Fig 1; Table 1). The compounds 3-geranyloxy-1,7-dihydroxyxanthone (**5**) and 7-geranyloxy-1,3-dihydroxyxanthone (**6**), isolated in small quantity, were identified in the chromatogram based on the mass spectral pattern of **7** and **8**. In contrast, garcinone B (**9**) was not detected, probably because of overlapping peaks and/or its low concentration.

Since propolis constituents are potential chemical markers for the plants that bees have visited for resin collection [34,43,44], suggestions for the botanical origin of propolis sample analysed were made. The revealing of resin sources is of interest as the appropriate plants contribute to high quality of propolis as well as to the bee colony health [4,45]. In general, the compounds identified by NMR and/or GC/MS data (Table 1) can be divided into three groups, according to the plants from which they have been previously isolated.

The first group includes the flavonoids **1**–**4** and liquiritigenin, and the stilbene resveratrol, among which the flavanes are well known constituents of the stem and red resin of *Dracaena cochinchinensis* (Agavaceae) [23,27,29,30,39,46]. The red resinous material, known as Chinese dragon’s blood, is a popular folk medicine used for treatment of fractures, wounds, stomach ulcers, etc. [23,47]. In fact, Dragon’s blood is a common name for the deep red resin/latex obtained from injured trunk and branches of plant species of four genera (*Dracaena*, *Croton*, *Pterocarpus* and *Daemonorops*) [47,48], amongst which *Dracaena* spp., and *D*. *cochinchinensis* plants in particular, are exclusively distributed in southern China, Vietnam and Laos [49]. It is interesting to note that Chinese Dragon’s blood has been regarded as an induced plant defence against pathogens and pests [48], which is also the role of propolis in the beehive. These findings gave us the chance to suppose a *Dracaena* spp., most probably *D*. *cochinchinensis*, as plant visited by *L*. *cacciae* for resin collection. This was somehow supported by the fact that the raw propolis and its 70% ethanol extract were characterized by a deep red color. In addition, *D*. *cochinchinensis* is the first monocotyledonous plant showed as a source of propolis resins.

Xanthones **5**–**9** and the tocopherol derivatives *δ*-tocotrienol and *β*/*γ*-tocotrienol form a second group of taxonomic markers. Recently, prenylated xanthones have been isolated from propolis of the stingless bees *Tetragonula leaviceps* [50] and *T*. *pagdeni* [51,52] collected in Thailand with proven botanical origin *Garcinia mangostana* (Hypericaceae) [52]. In *L*. *cacciae* propolis, however, together with *α*-mangostin (**8**) and garcinone B (**9**), xanthones bearing a geranyl(oxy) group were isolated, and their simultaneous occurrence was found to be characteristic for *Cratoxylum cochinchinense* (Hypericaceae), a tropical medicinal plant distributed in Southeast Asia [53]. Numerous investigations have revealed that its stem, resin and green fruits are rich source of xanthones with antimicrobial, cytotoxic and antimalarial activities [23,54–56], and a source of vitamin E like compounds [57]. The above-mentioned data provide an evidence for the contribution of *C*. *cochinchinense* as a second plant source of the sample analysed. Moreover, *C*. *cochinchinense* seems to be the plant which is preferred by *Lisotrigona* bees’ species as it is the botanical source of propolis collected by *L*. *furva* in the same Vietnamese location [16].

The third group of compounds is the combination of the phenolic lipids resorcinols **12a-g** and anacardic acids together with the triterpenes, which are found in propolis containing resin of *M*. *indica*, as mentioned above. *M*. *indica* propolis type has been revealed for honey bee propolis in many regions of Asia, such as Oman [37], Thailand [38], Myanmar [35] and Indonesia [36]. Interestingly, *M*. *indica* was also suggested to be a source of Vietnamese propolis from the stingless bee *Trigona minor* [58,59].

The results showed that the propolis sample analysed is of triple botanical origin. Mixed propolis types have also been established for *Apis mellifera* propolis collected in different geographical regions [37,60,61].

### Antimicrobial activity

The crude extract and some of the isolated compounds were evaluated *in vitro* for antimicrobial potency against *S*. *aureus*, *E*. *coli*, and *C*. *albicans*. The results (Table 3) showed that the compounds inhibited all three microorganisms, while the crude extract was inactive against *C*. *albicans*. For *α*-mangostin (**8**), significant activity was observed against *S*. *aureus* with MIC and MBC 0.31 μg/ml, followed by 7,4’-dihydroxy-5-methoxy-8-methylflavane (**1**) with MIC 78 μg/ml and MBC 156 μg/ml. The other constituents displayed low activity, as **4**, **7, 10** and **12a-g** inhibited the Gram negative bacteria *E*. *coli* at lower concentration.

**Table 3:** Antimicrobial activities of 70% ethanol extract and isolated compounds.

Sample	*S*. *aureus* SAIM 209		*E*. *coli* SAIM WF+		*C*. *albicans* SAIM 562	
	MIC	MBC	MIC	MBC	MIC	MBC
	μg/ml					
70% ethanol extract	156	156	156	156	NA	NA
**1**	78	156	156	156	156	156
**2**	156	313	156	156	156	156
**3**	156	156	156	156	156	313
**4**	313	625	156	156	313	625
**7**	313	625	156	156	313	625
**8**	0.31	0.31	156	156	156	156
**10**	313	313	156	156	313	313
**12**_**a-g**_	313	625	156	156	313	625
Gentamicin^a^	0.03	0.03	0.5	0.5	NT	NT
Amphotericin B^a^	NT	NT	NT	NT	0.125	0.125

MIC, minimal inhibitory concentration; MBC, minimal bactericidal concentration; SAIM, collection of the Stephan Angeloff Institute of Microbiology. NA, no activity (> 2.5 mg/ml); NT, not tested

^a^Positive control.

It is interesting to note that *α*-mangostin (**8**) and garcinone B (**9**) are the major compounds contributing to the antibacterial activity of Thai stingless bee propolis [49], and **8** is one of the most active principles in *C*. *cochinchinense* [23]. Boonnak et al. [23] also found a significantly greater antibacterial effect for *α*-mangostin as compared to cochinchinone A, as well as a higher potency for the mixture of geranyloxy xanthones **5** and **6** in comparison to that of the individual compounds against a panel of Gram positive bacteria.

### Free radical scavenging activity

DPPH radical scavenging activity was tested for the crude extract and isolated phenols **1**–**9**, and **12a-g**. All tested samples, except for compound **4**, were inactive (IC_50_ >1000 μg/ml). The 10,11-dihydroxydracaenone C (**4**) displayed good antioxidant ability with IC_50_ 116.0 μg/ml, vs. IC_50_ 69.3 μg/ml of the positive control caffeic acid. Moreover, **4** is the only isolated component bearing *ortho*-hydroxyl groups, which is in agreement with the fact that *ortho*-hydroxyl phenols exhibit enhanced antioxidant activity [62]. The lack of antioxidant activity of the crude extract could be explained by the findings that **4** is also the only *ortho*-hydroxylated molecule identified in the complex mixture of compounds. No ability to scavenge the free DPPH radicals for the homoisoflavans and xanthones has been observed by several research groups [51,62,63].

## Conclusion

In the present article, a phytochemical study of propolis produced by the stingless bee *Lisotrigona cacciae* was described for the first time. The results add new knowledge in the field of propolis research in terms of new constituents and a new plant source. The study also reveals that the propolis of *L*. *cacciae*, and stingless bee propolis in general, is a valuable product as well as a promising source of biologically active compounds.

## Supporting information

S1 Fig Flow chart of the sample analysis. (PDF)

S2 Fig ^1^H, ^13^C, DEPT 135, HSQC, HMBC and NOESY NMR spectra of compound 1 in CD_3_OD. (PDF)

S3 Fig ^1^H, HSQC and HMBC NMR spectra of compound 2 in CD_3_OD. (PDF)

S4 Fig ^1^H, HSQC, HMBC and NOESY NMR spectra of compound 3 in CD_3_OD. (PDF)

S5 Fig ^1^H and HSQC NMR spectra of compound 4 in DMSO-d_6_. (PDF)

S6 Fig ^1^H NMR spectrum of a mixture of 5 and 6 in CD_3_OD. (PDF)

S7 Fig ^1^H, ^13^C, HSQC and HMBC NMR spectra of compound 7 in CDCl_3_. (PDF)

S8 Fig ^1^H NMR spectrum of compound 8 in CD_3_OD. (PDF)

S9 Fig ^1^H NMR spectrum of compound 9 in CD_3_OD. (PDF)

S10 Fig ^1^H NMR spectrum of compound 10 in CDCl_3_. (PDF)

S11 Fig ^1^H NMR spectrum of compound 11 in CDCl_3_. (PDF)

S12 Fig ^1^H NMR spectrum of a mixture 12 in CDCl_3_. (PDF)
