# Supplementary figures and images for: Phytochemical analysis of Vietnamese propolis produced by the stingless bee Lisotrigona cacciae
Source: PLoS One. 2019 Apr 24;14(4):e0216074. doi: 10.1371/journal.pone.0216074 (PMC6481864; doi:10.1371/journal.pone.0216074)

**S1 Fig. Flow chart of the sample analysis.**

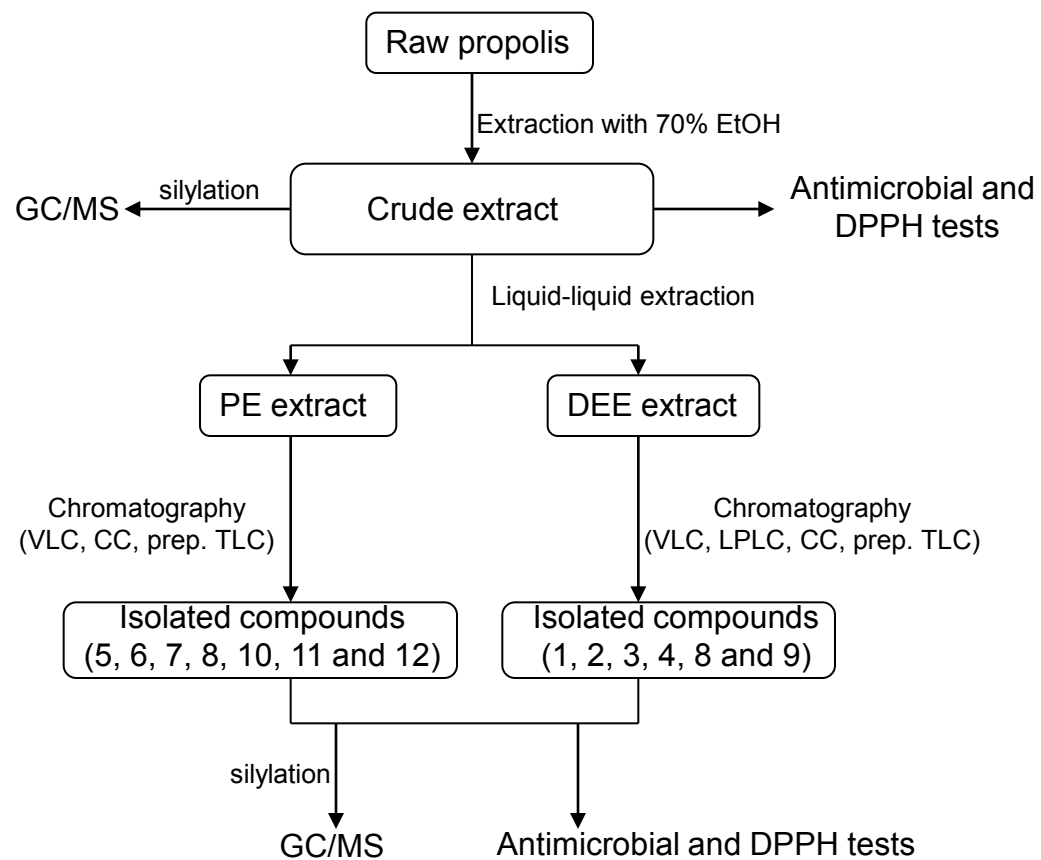

Supplement: S1 Fig — (PDF) [file pone.0216074.s001.pdf]

**S2 Fig.  $^1\text{H}$ ,  $^{13}\text{C}$ , DEPT 135, HSQC, HMBC and NOESY NMR spectra of compound 1 in  $\text{CD}_3\text{OD}$ .**

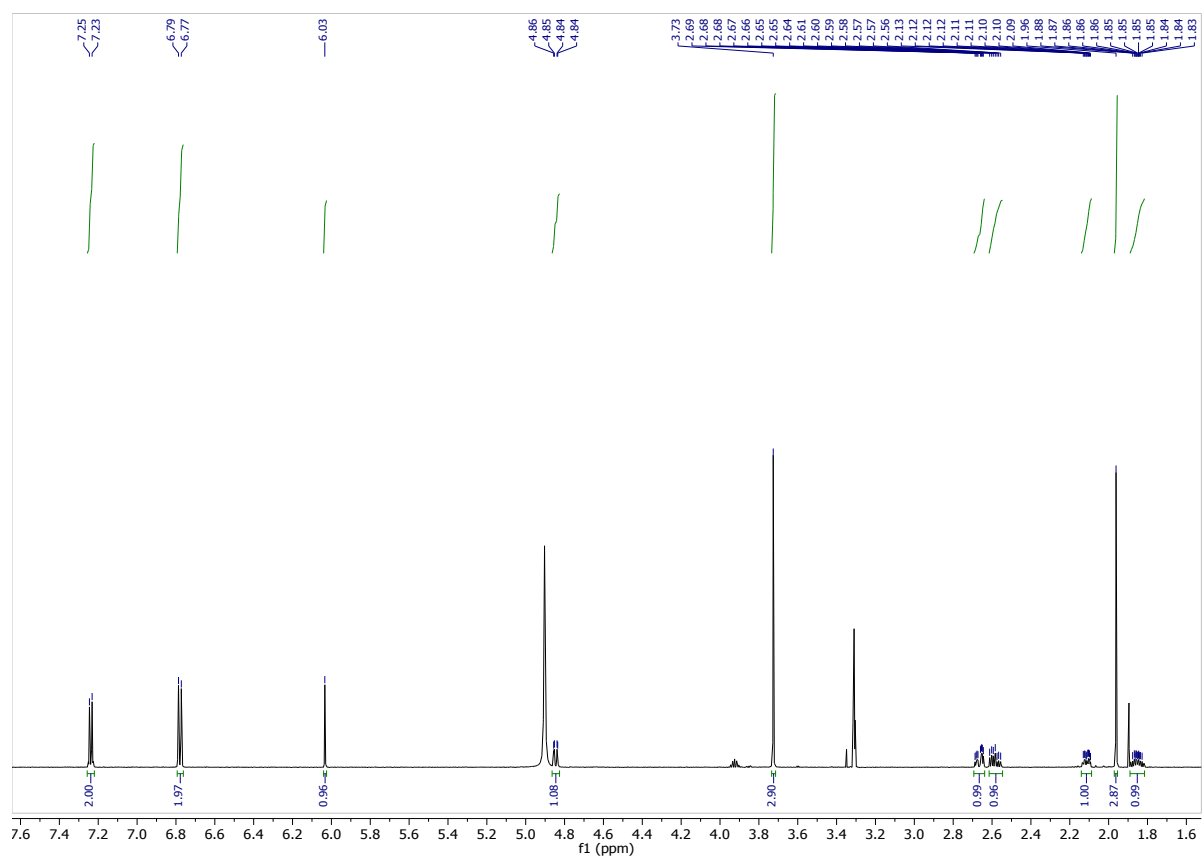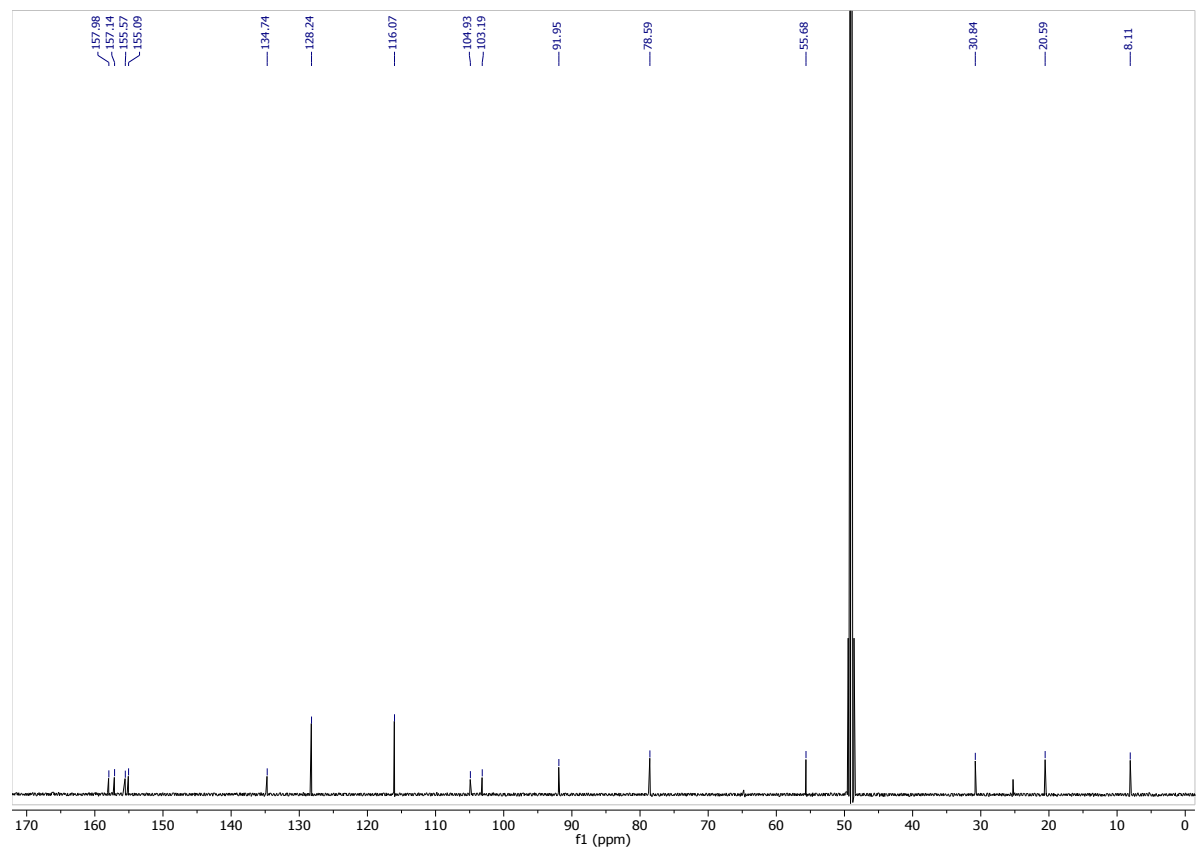

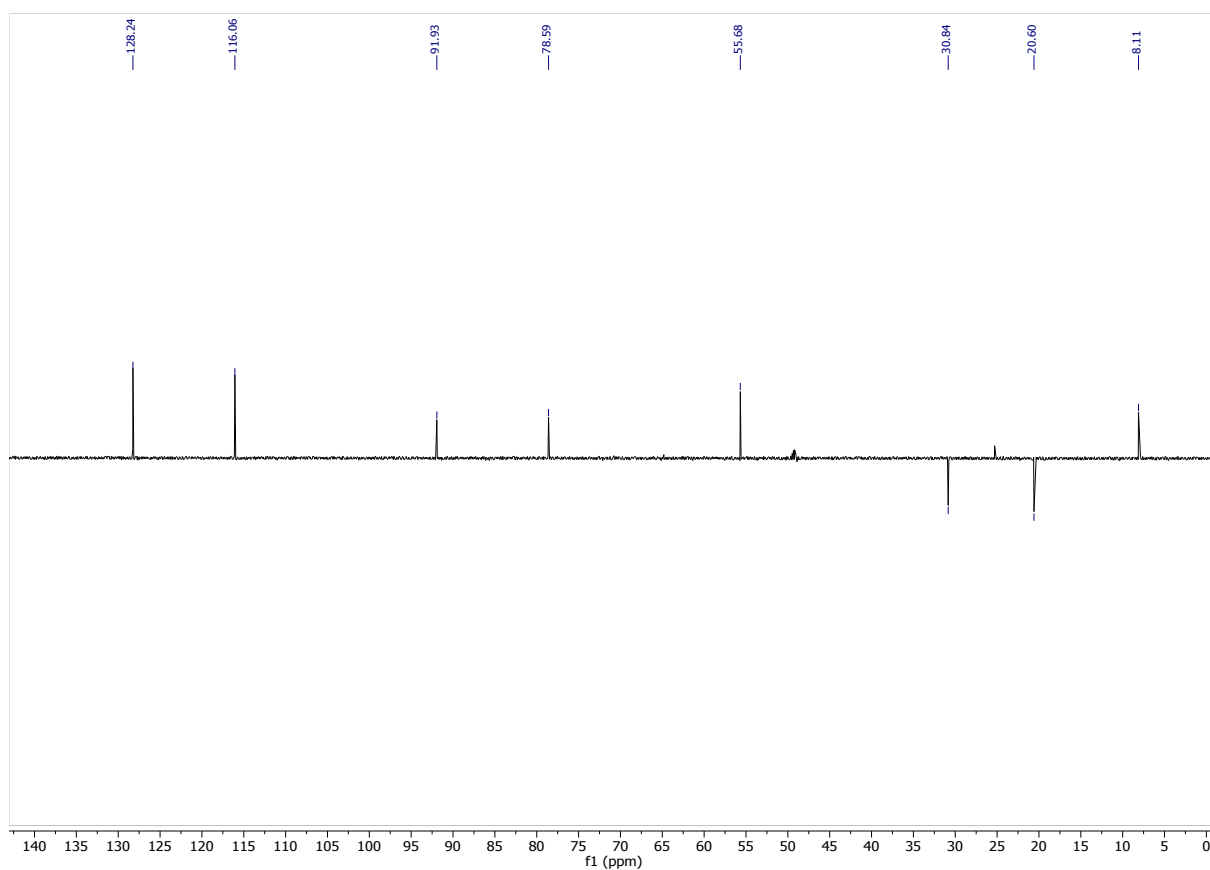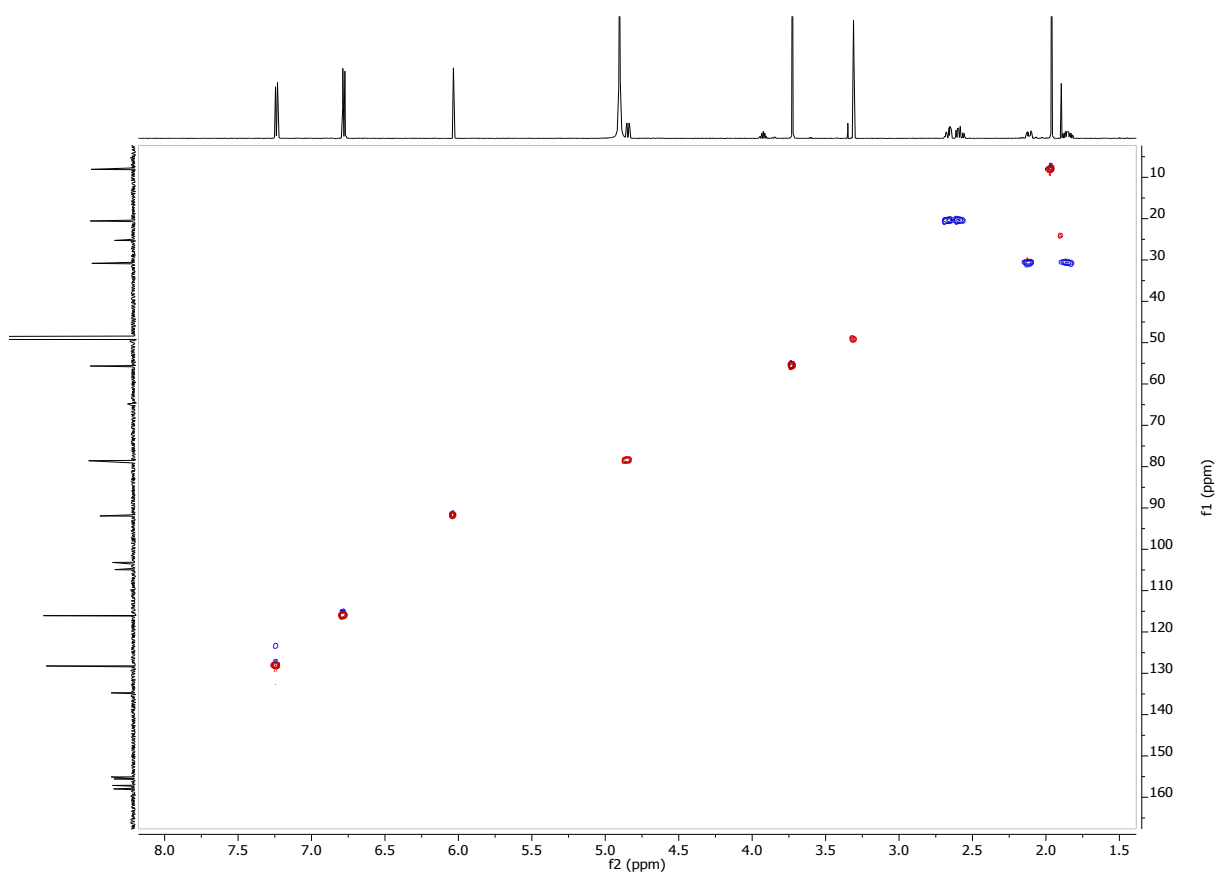

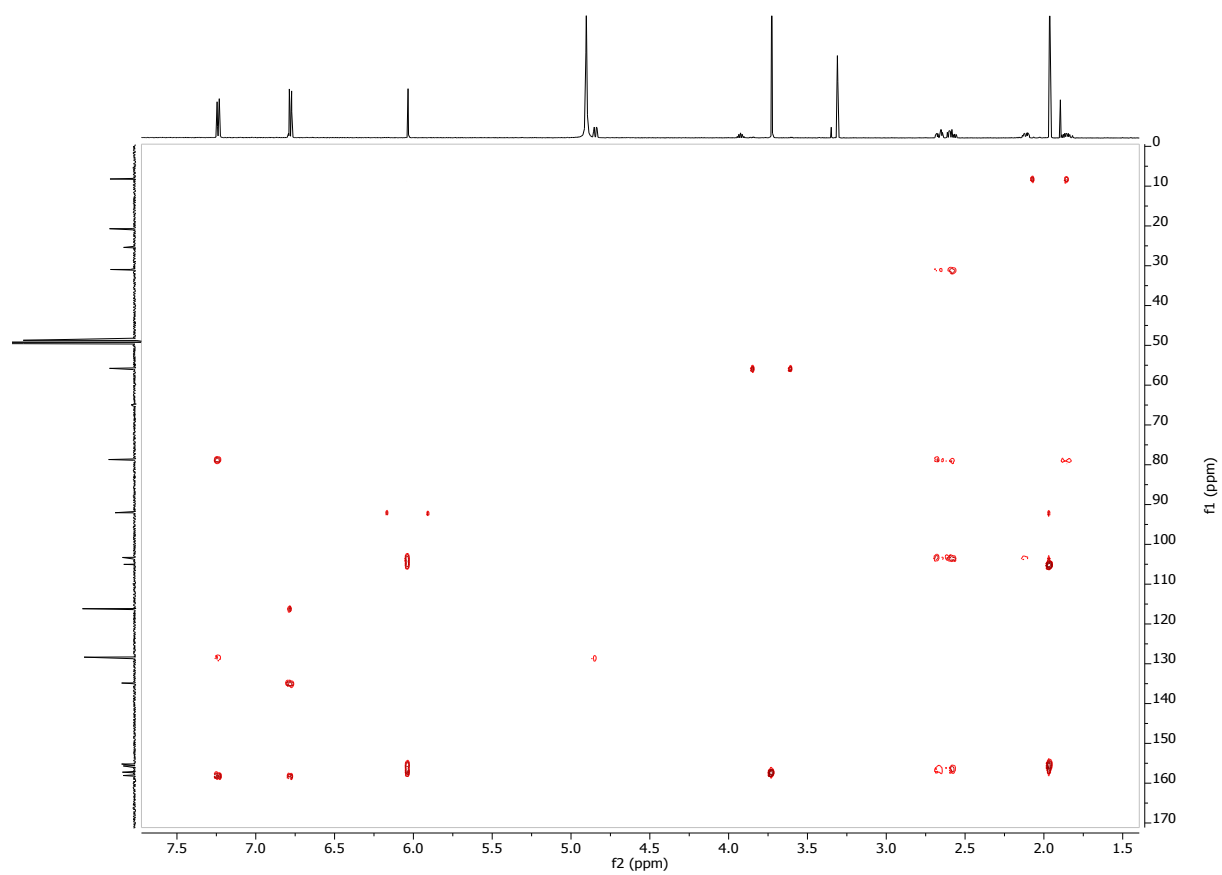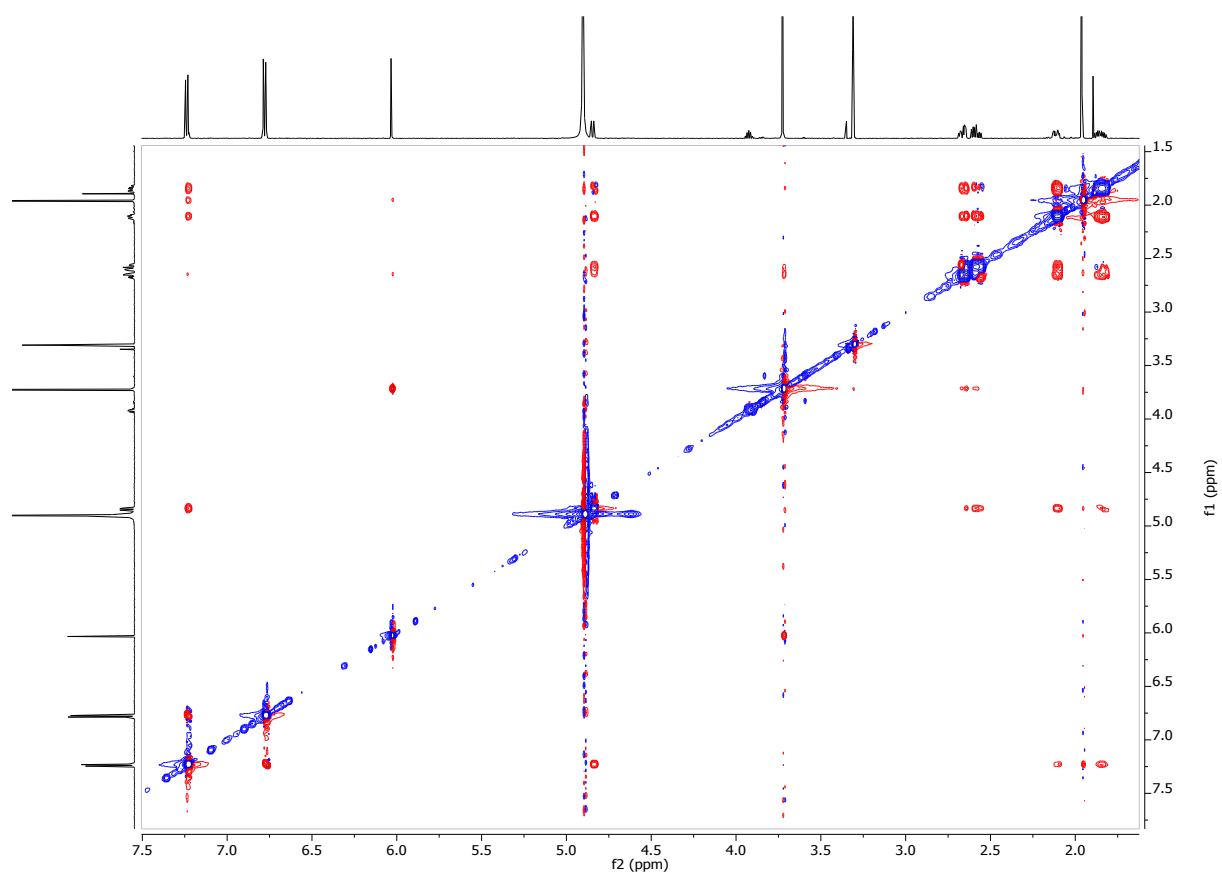

Supplement: S2 Fig — (PDF) [file pone.0216074.s002.pdf]

**S3 Fig.**  $^1\text{H}$ , HSQC and HMBC NMR spectra of compound 2 in  $\text{CD}_3\text{OD}$ .

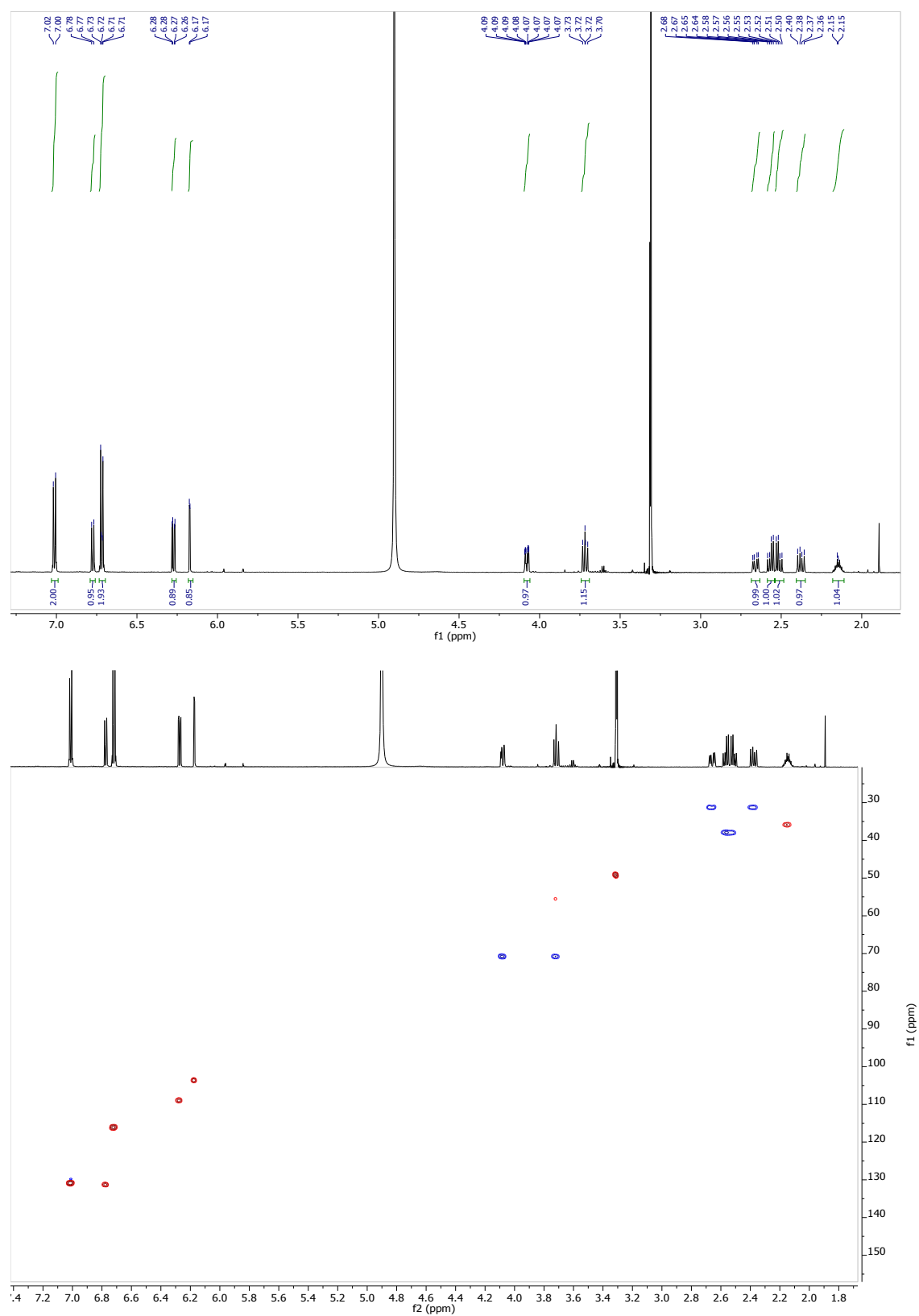

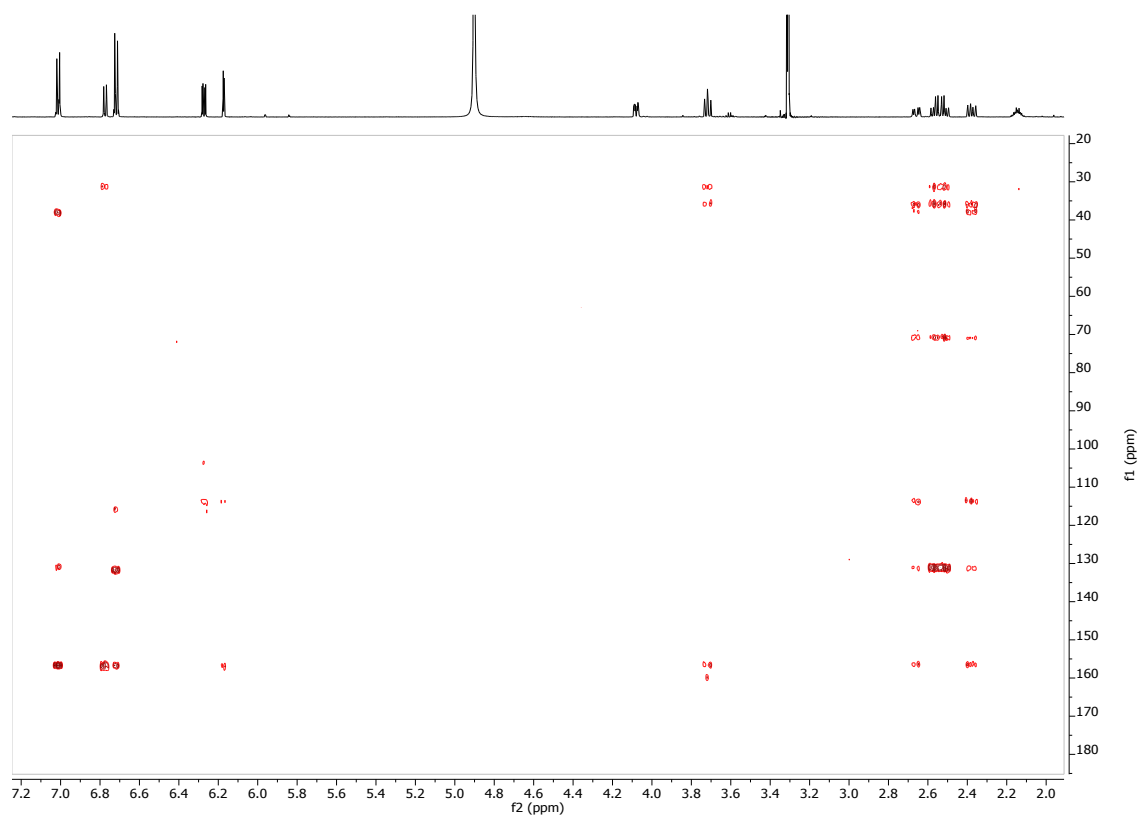

Supplement: S3 Fig — (PDF) [file pone.0216074.s003.pdf]

**S4 Fig.**  $^1\text{H}$ , HSQC, HMBC and NOESY NMR spectra of compound 3 in  $\text{CD}_3\text{OD}$ .

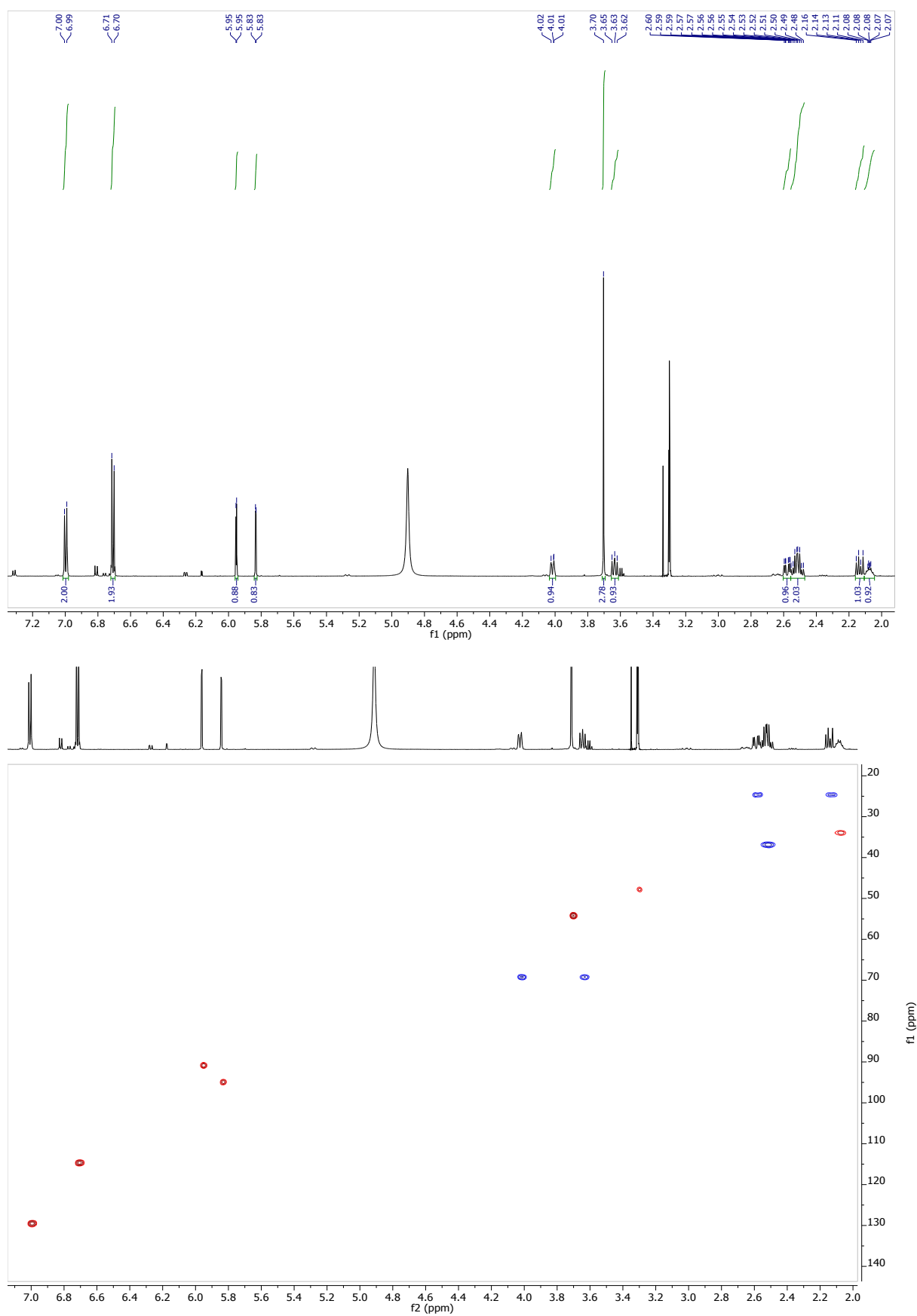

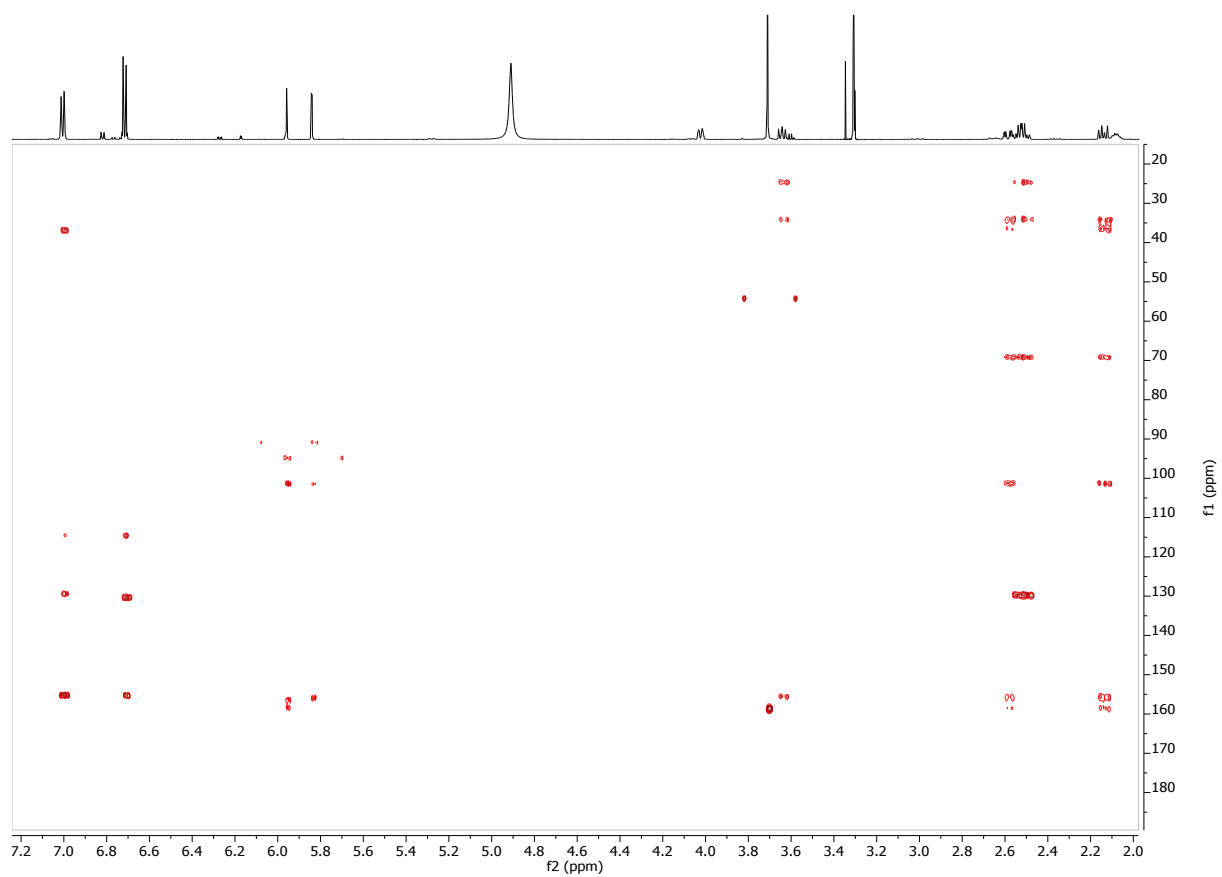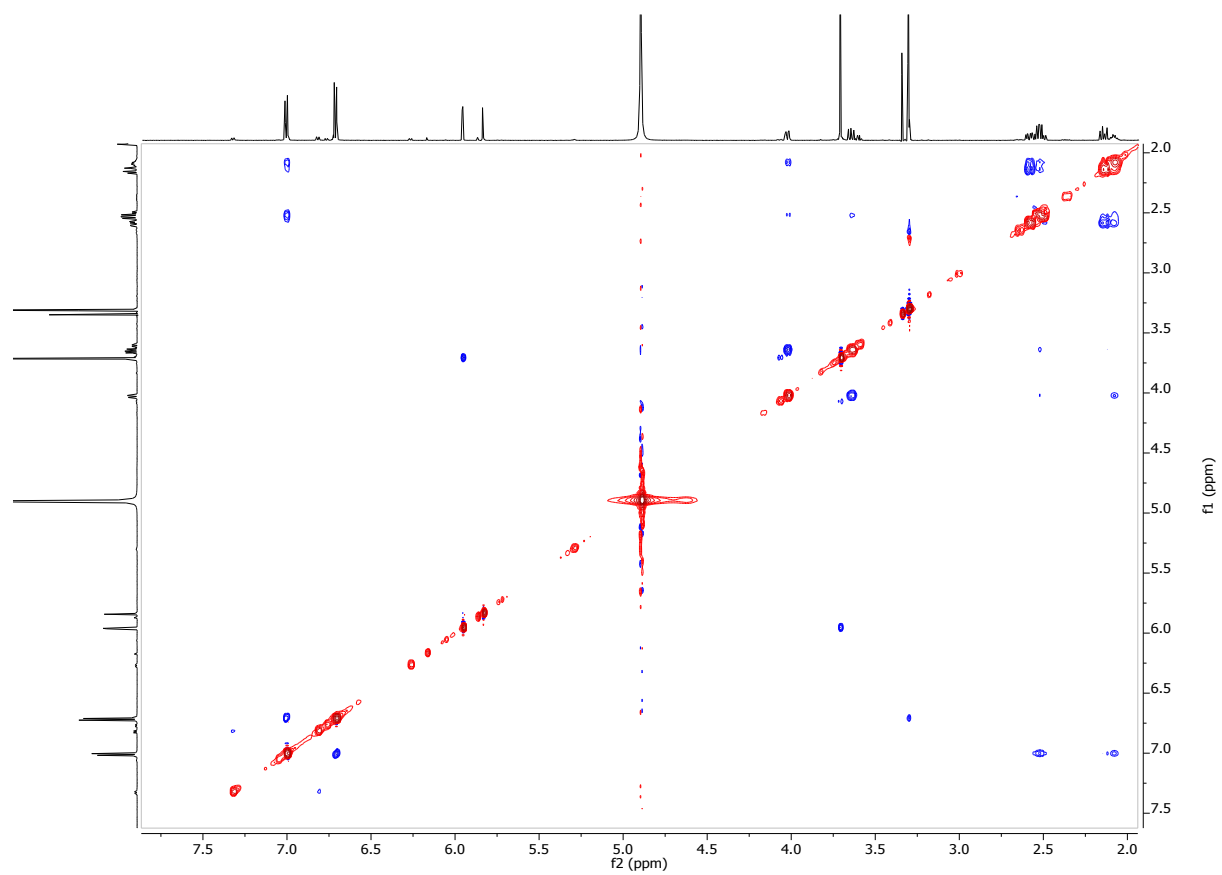

Supplement: S4 Fig — (PDF) [file pone.0216074.s004.pdf]

**S5 Fig.**  $^1\text{H}$  and HSQC spectra of compound 4 in  $\text{DMSO-d}_6$ .

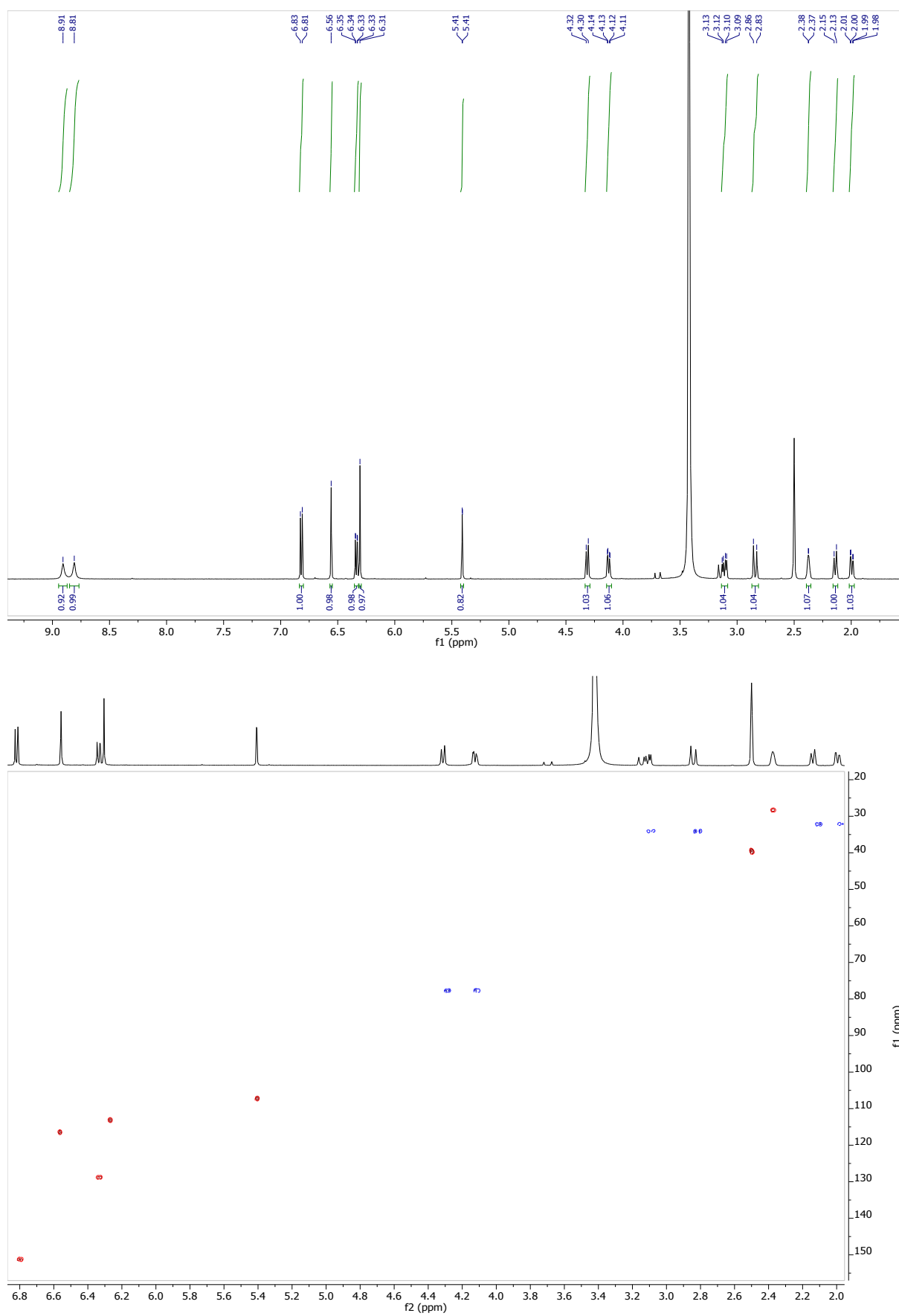

Supplement: S5 Fig — (PDF) [file pone.0216074.s005.pdf]

**S6 Fig.**  $^1\text{H}$  NMR spectrum of a mixture of compounds **5** and **6** in  $\text{CD}_3\text{OD}$ .

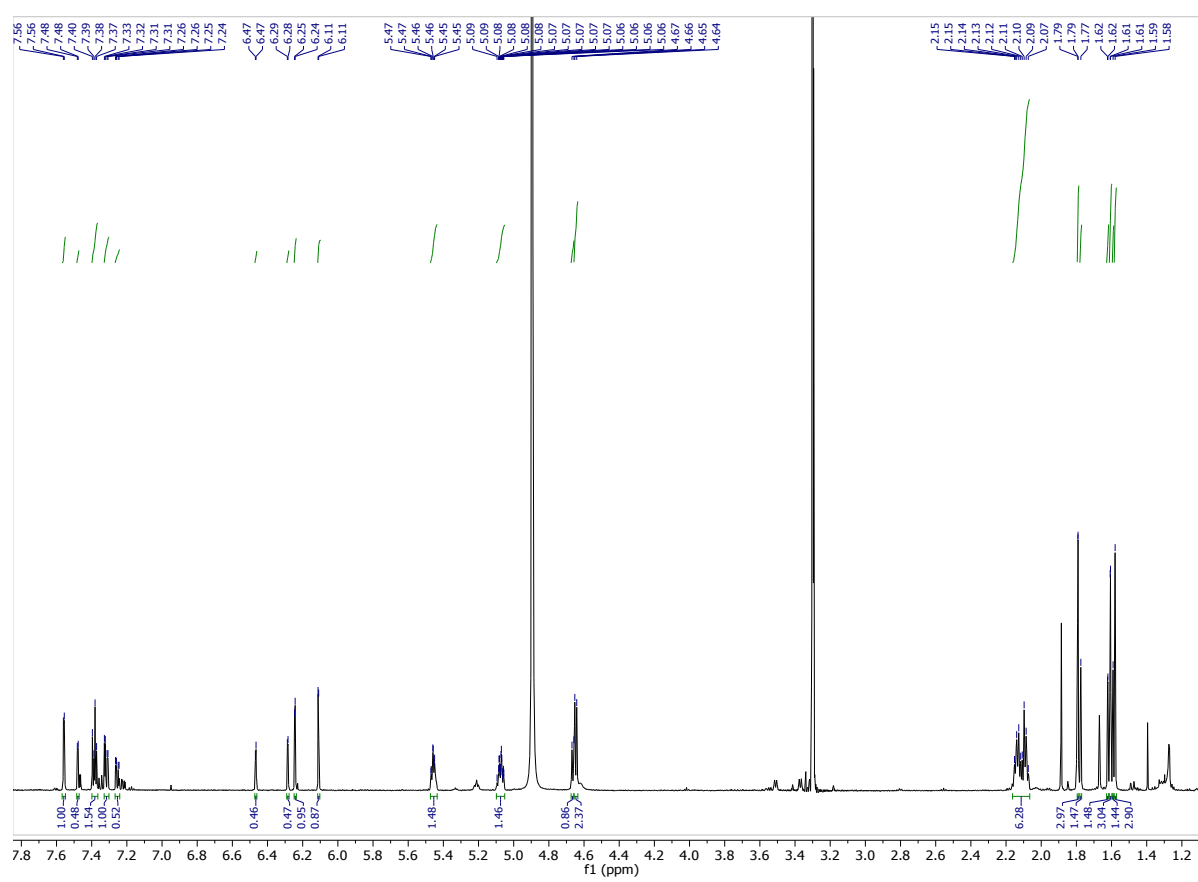

Supplement: S6 Fig — (PDF) [file pone.0216074.s006.pdf]

**S7 Fig.  $^1\text{H}$ ,  $^{13}\text{C}$ , HSQC and HMBC NMR spectra of compound 7 in  $\text{CDCl}_3$ .**

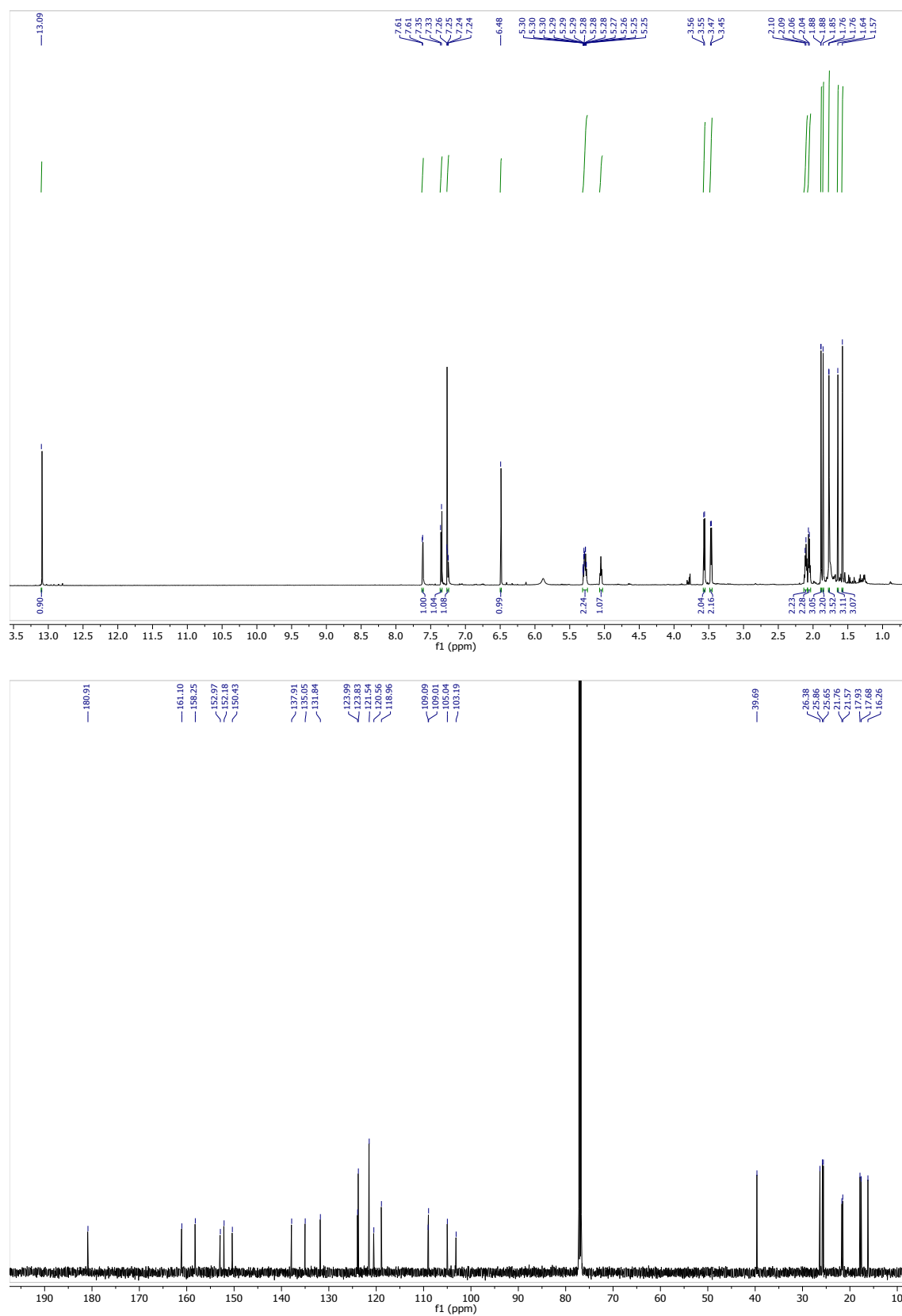

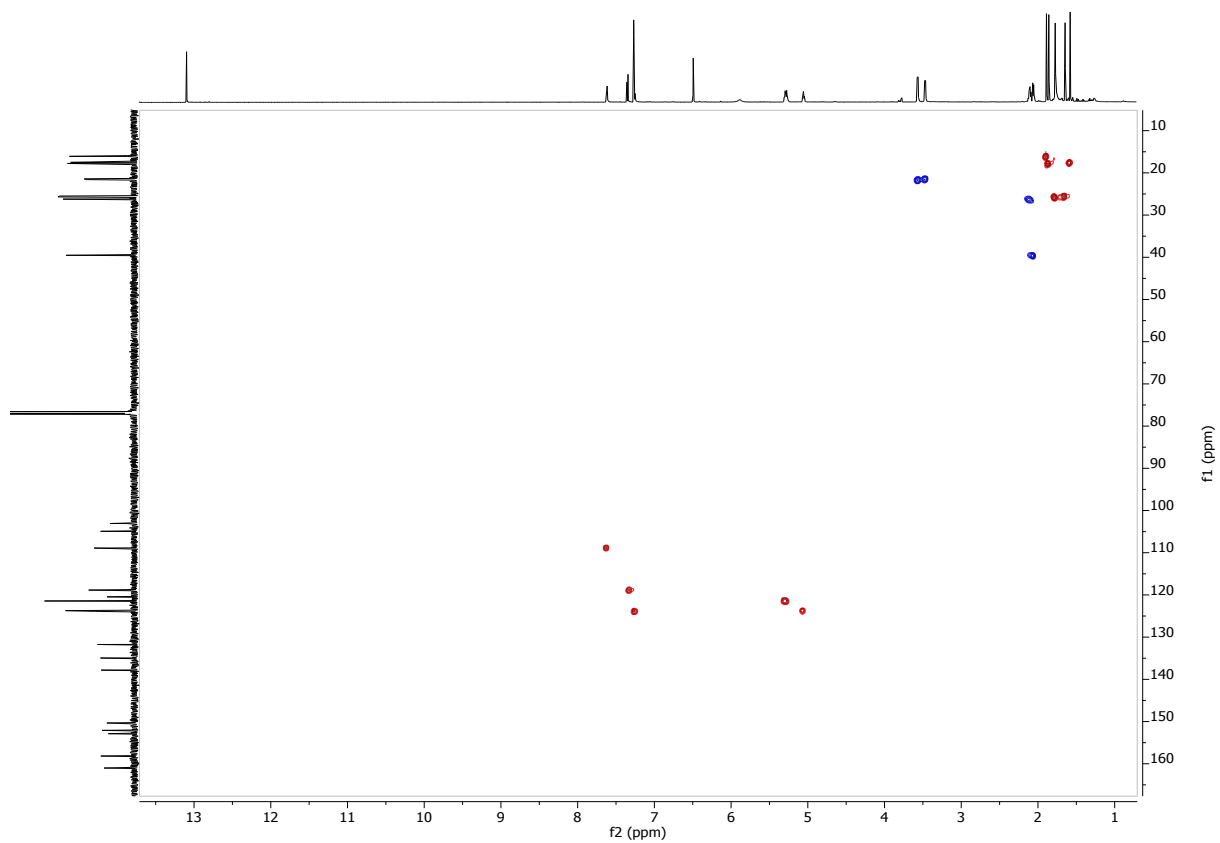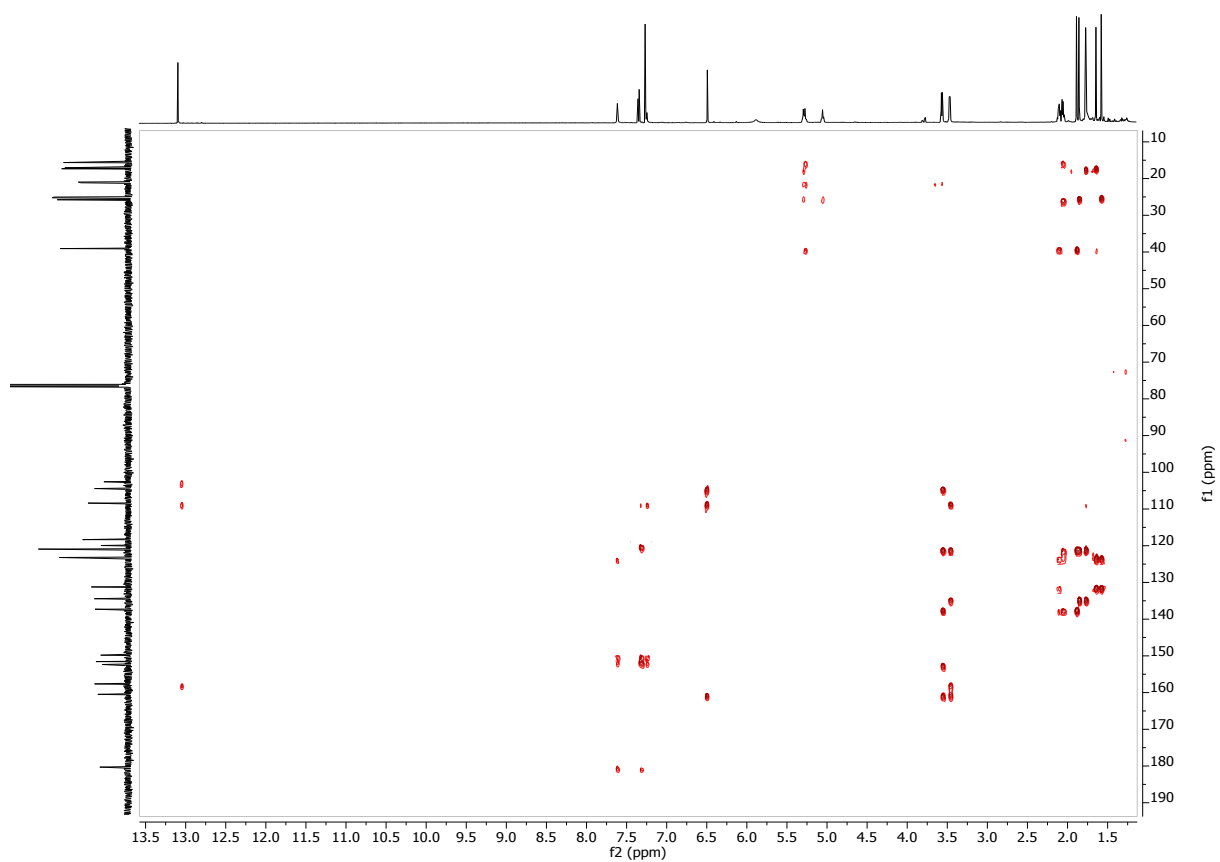

Supplement: S7 Fig — (PDF) [file pone.0216074.s007.pdf]

**S8 Fig.**  $^1\text{H}$  NMR spectrum of compound 8 in  $\text{CD}_3\text{OD}$ .

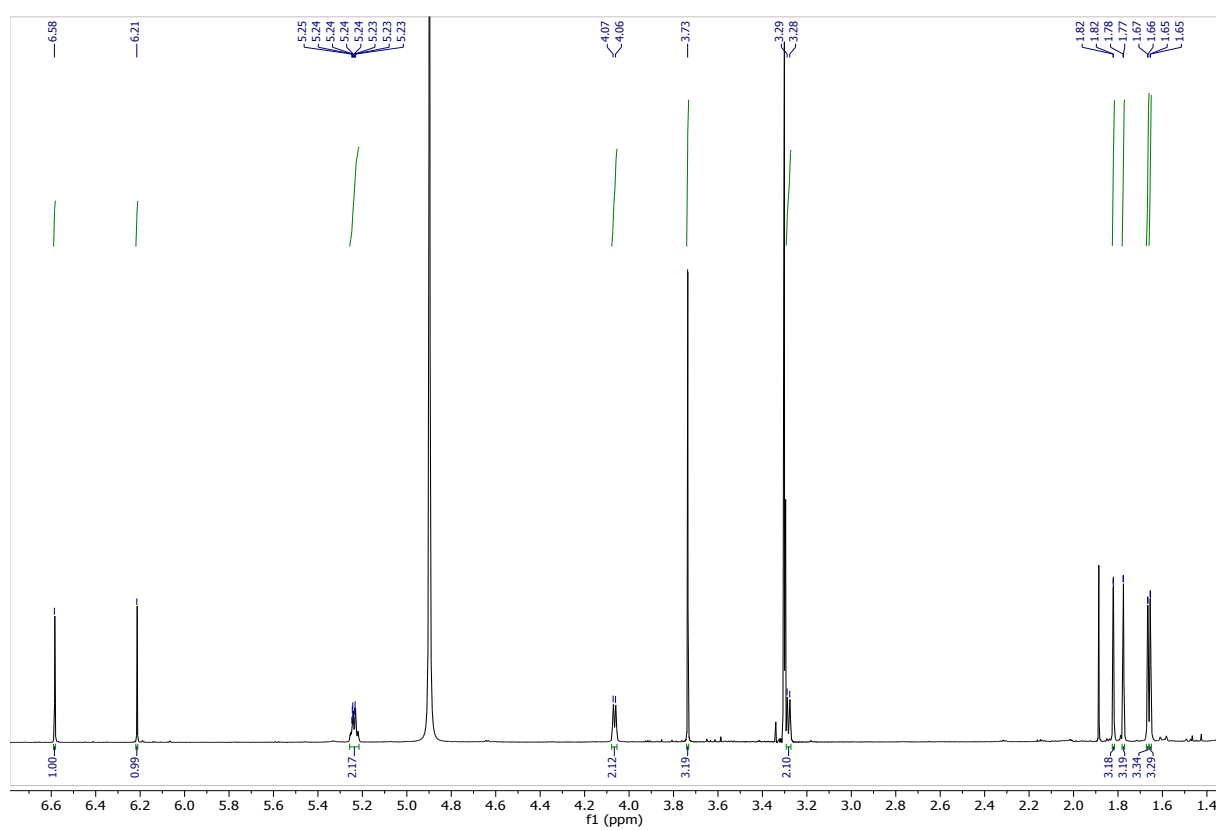

Supplement: S8 Fig — (PDF) [file pone.0216074.s008.pdf]

**S9 Fig.**  $^1\text{H}$  NMR spectrum of compound 9 in  $\text{CD}_3\text{OD}$ .

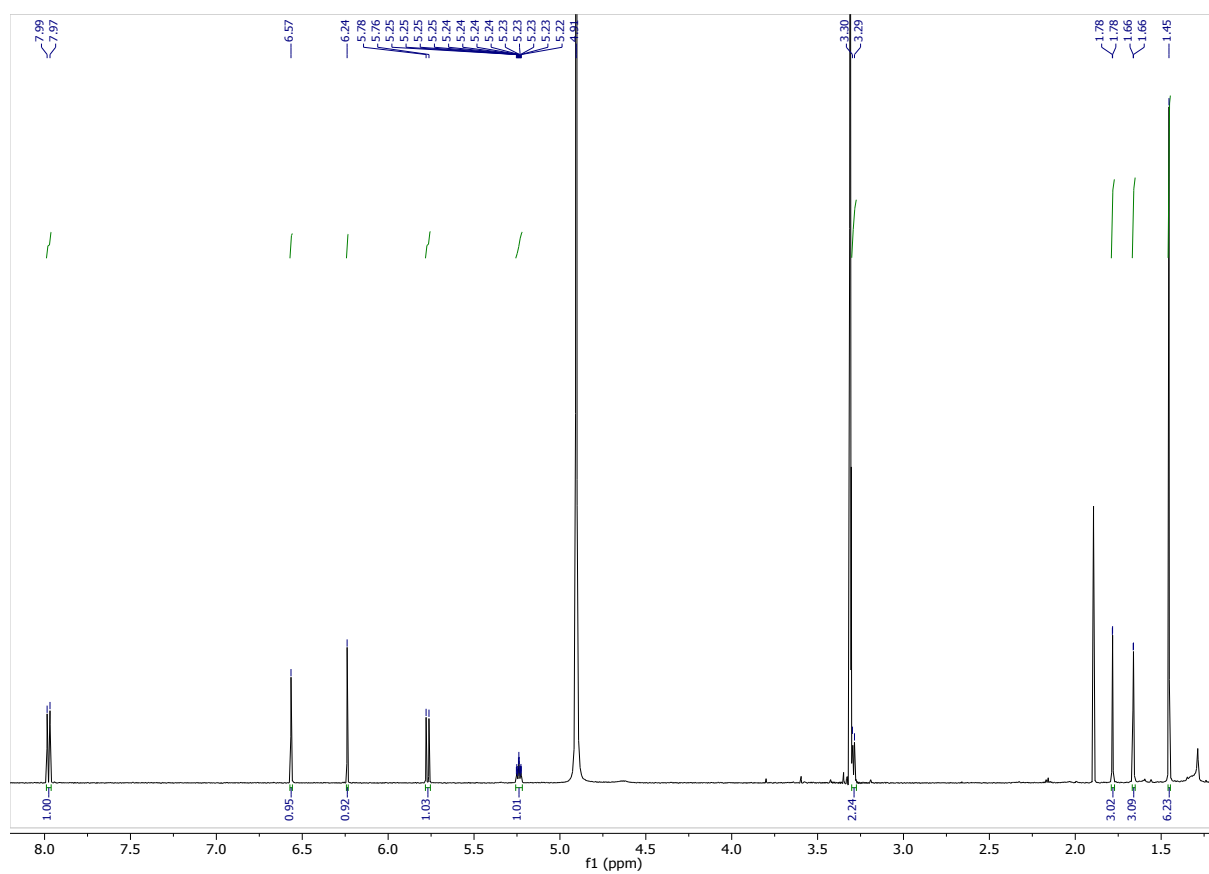

Supplement: S9 Fig — (PDF) [file pone.0216074.s009.pdf]

**S10 Fig.**  $^1\text{H}$  NMR spectrum of compound 10 in  $\text{CDCl}_3$ .

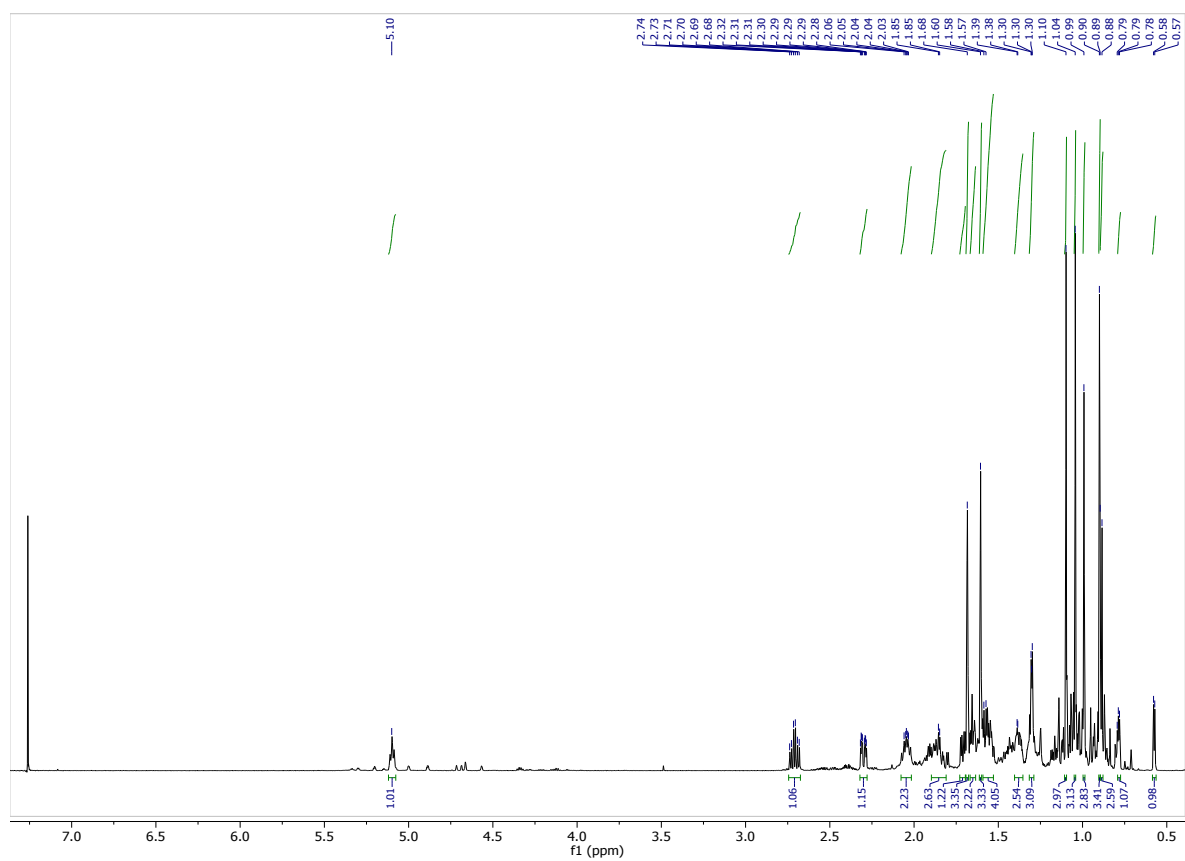

Supplement: S10 Fig — (PDF) [file pone.0216074.s010.pdf]

S11 Fig.  $^1\text{H}$  NMR spectrum of compound 11 in  $\text{CDCl}_3$ .

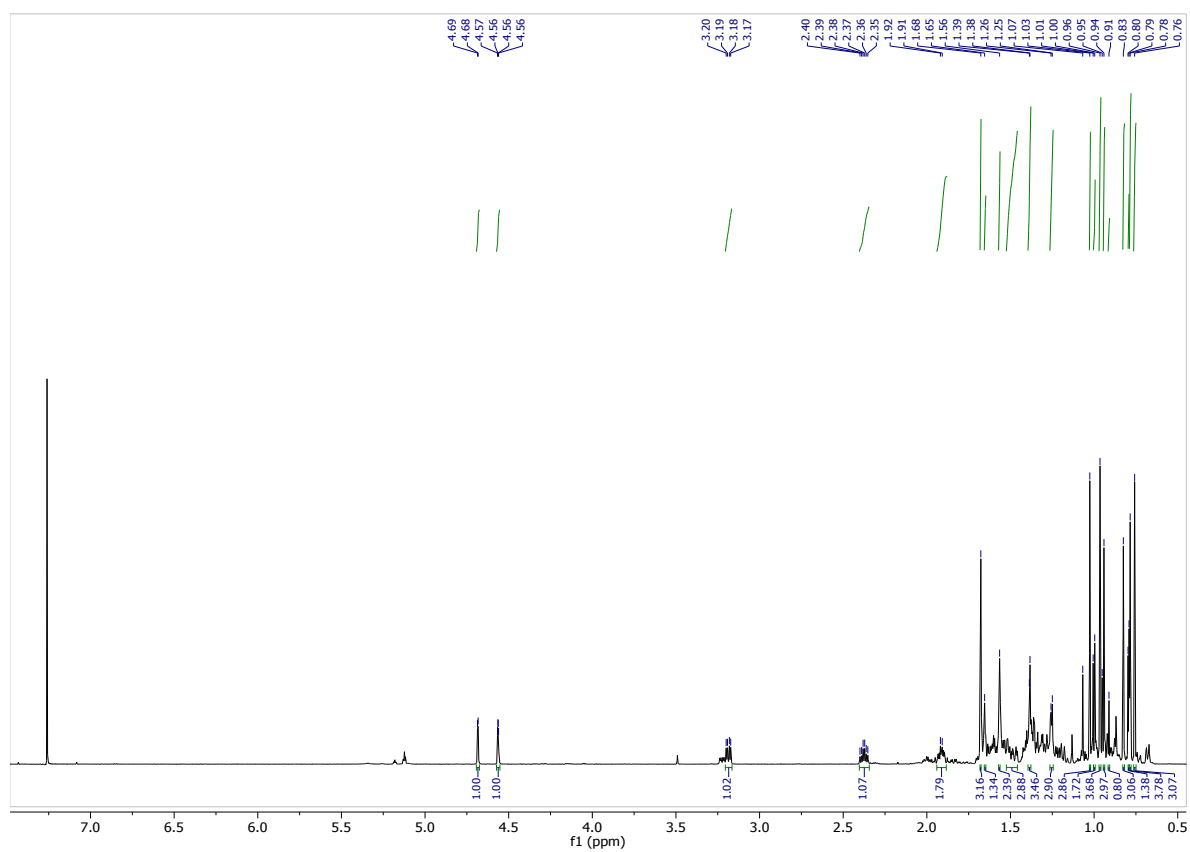

Supplement: S11 Fig — (PDF) [file pone.0216074.s011.pdf]

**S12 Fig.**  $^1\text{H}$  NMR spectrum of a mixture of 12 in  $\text{CDCl}_3$ .

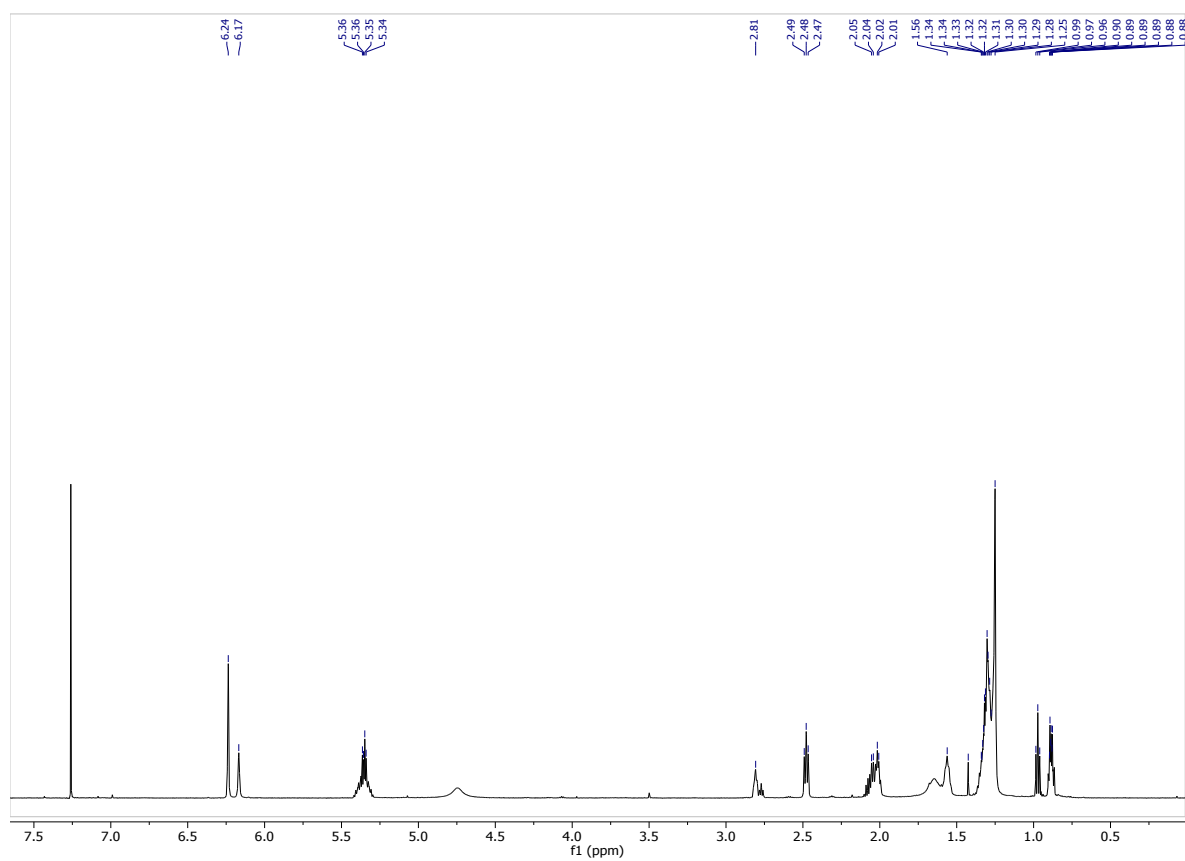

Supplement: S12 Fig — (PDF) [file pone.0216074.s012.pdf]
